# Electrospun nanofiber membrane of chitosan/polyvinyl alcohol embedded with DOX-loaded V-MOFs for controlled drug release and multifunctional biological activity

**DOI:** 10.1039/d6ra01018k

**Published:** 2026-04-01

**Authors:** Ahlem Guesmi, Naoufel Ben Hamadi, Wesam Abd El-Fattah, Mohamed G. El-Desouky, Ashraf A. El-Bindary

**Affiliations:** a Chemistry Department, College of Science, Imam Mohammad Ibn Saud Islamic University (IMSIU) Riyadh 11623 Saudi Arabia; b Egyptian Propylene and Polypropylene Company Port Said 42511 Egypt; c Chemistry Department, Faculty of Science, Damietta University Damietta 34517 Egypt abindary@du.edu.eg; d Health Sciences Research Center (HSRC), Deanship of Scientific Research, Imam Mohammad Ibn Saud Islamic University (IMSIU) Riyadh 13317 Saudi Arabia

## Abstract

The integration of doxorubicin-loaded vanadium metal–organic frameworks (DOX@V-MOFs) into chitosan/polyvinyl alcohol (CS/PVA) electrospun nanofiber membranes has resulted in a novel multifunctional, stimuli-responsive drug delivery system. This hybrid platform was synthesized *via* one-step electrospinning and characterized thoroughly using FT-IR, XRD, SEM, XPS, EDX, and N_2_ adsorption–desorption analyses to confirm successful drug encapsulation with the structural integrity of the support material being maintained. The resultant membrane demonstrated a dual-responsive mechanism towards pH and temperature stimuli that significantly enhanced drug release under simulated tumor conditions at an acidic pH of 5.0 and elevated temperatures from 37 to 42 °C. The release kinetics followed zero-order, Higuchi, and Korsmeyer-Peppas models indicating the dominant role of diffusion-controlled mechanisms in the release processes. Extremely potent cytotoxicity against various cancer cell lines was displayed by the DOX@V-MOF membranes including models for liver cancer (HepG-2) and breast cancer (MCF-7) together with high antioxidant as well as antibacterial properties against *Candida albicans*, *Staphylococcus aureus*, and *Escherichia coli*. Optimization through a Box-Behnken design revealed that pH, temperature, and time are significant parameters controlling the dynamics of drug release. These results support the hypothesis that DOX@V-MOF nanofibers may serve as a new mechanism for controlled drug release in localized cancer therapy along with multiple biomedical applications requiring integrated anticancer, antioxidant, and antimicrobial properties.

## Abstract 2

The integration of doxorubicin-loaded vanadium metal–organic frameworks (DOX@V-MOFs) into chitosan/polyvinyl alcohol (CS/PVA) electrospun nanofiber membranes has resulted in a novel multifunctional, stimuli-responsive drug delivery system.

## Graphical abstract

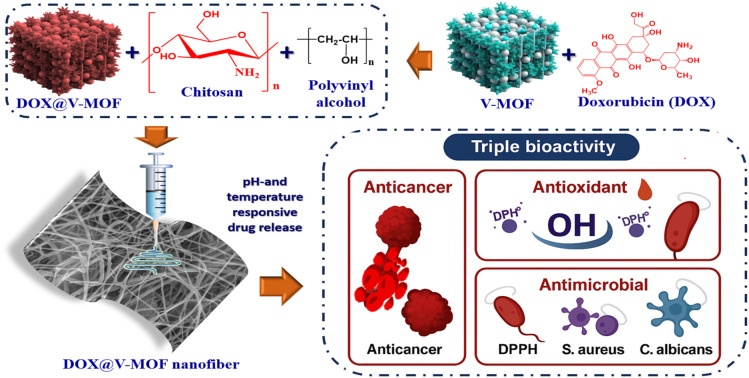

## 1. Introduction

Recent advances in nanotechnology and materials science have significantly influenced biomedical research, especially concerning novel strategies for drug delivery.^1^ Conventional chemotherapy is frequently limited by systemic toxicity, poor bioavailability, and unspecific drug distribution. This results in side effects and low efficacy. Therefore, significant strides have been made in the design of targeted and stimuli-receptive drug delivery schemes meant to increase the accumulation of therapeutic agents at the disease site while reducing their exposure to healthy tissues. Nanostructured materials with tunable physicochemical properties are now broadly applied in this area; hence enabling researchers to create a drug delivery system that can answer to environmental incentives like temperature, pH, and enzymatic activity. These innovations bear enormous potential for cancer treatment since they permit amplified active targeting based on the precise biological appearance of the tumor microenvironment for selective drug release.^2^

Metal–organic frameworks are crystalline compounds formed by the coordination of metal ions or clusters with organic linkers to create extended porous structures. Their versatility allows for the selective alteration of chemical composition and pore structure to fulfill specific requirements in the biomedical field. Properties like large surface areas, tunable pore sizes, high drug loading capacities, and adjustable surface characteristics have made MOFs very popular for nanocarrier development.^3^ A special family of MOFs with intriguing redox characteristics that can be used in treatment are V-MOFs. The presence of vanadium is responsible for potential antioxidant and anticancer activities, as well as enhancing the overall performance of a drug delivery scheme. This article will review the application of V-MOFs as functional materials and carriers for drugs to improve the efficacy of therapies.^4^

Doxorubicin, also known as DOX, is a commonly used drug in chemotherapy. It has strong effects against different cancers, such as those of the breast, liver, and skin ^5^. Its healing use is limited by issues of heart toxicity that depend on the dose, and the creation of resistance to many drugs. To get around these problems, putting DOX inside a tiny carrier system, especially metal–organic frameworks (MOFs), has been looked at.^6^ This approach has previously been reported to successfully enhance drug solubility, provide protection from premature degradation, and facilitate a more controlled release mechanism. Moreover, the application of this approach can potentially address the issue of drug resistance by maintaining the drug concentration at the tumor site above the therapeutic threshold for an extended duration. In this work, V-MOFs have been used as drug carriers for DOX, capitalizing on their porosity and redox-active features to achieve highly targeted and controlled delivery of the drug that may enhance the cytotoxic activity of DOX with lower systemic toxicity.^7^

To improve the drug delivery systems based on MOFs and their compatibility with biological environments, it is necessary to encapsulate them in a polymeric matrix that has both biodegradability and biocompatibility.^8^ Due of their high ratios of surface area to volume, ease of modifying their surfaces, and similarity to the extracellular matrix, membranes made from electrospun nanofibers have become popular as efficient carriers for drug delivery.^9^ Chitosan (CS) is a bio polysaccharide obtained from natural sources. It has inherent antibacterial, antioxidant, and wound healing properties that make it an attractive candidate for different biomedical applications. Polyvinyl alcohol (PVA) is a synthetic polymer that can enhance the properties of CS by improving its mechanical strength and spinnability. The combination of CS and PVA to form nanofibers results in a hybrid material that synergizes the advantageous properties of both components. This work reports on the electrospinning fabrication of a nanofiber membrane from CS blended with PVA doped with doxorubicin-loaded V-MOFs for application as a multifunctional smart drug delivery system.^10^

Recently, there has been significant attention given to MOF-depending on nanocarriers for the controlled therapy of doxorubicin (DOX). Multiple studies have reported on MOF-polymer hybrid systems designed to attain high drug encapsulation efficacy and continued release profiles through diffusion-controlled mechanisms.^11,12^ Incorporation of MOFs in biopolymeric matrices like CS or PVA has provided better structural stability and environmental response to physiological conditions. Because of their high surface area and adjustable porosity, which enable effective drug loading and regulated release behavior, electrospun nanofiber-based platforms with integrated MOFs are likewise receiving more and more attention. Most of these systems still have limitations such as burst release effects, inadequate response to tumor-like acidic environments, and limited modulation of release kinetics under different physiological conditions. This therefore emphasizes the need for multifunctional MOF-based nanofiber membranes that would offer better pH- and temperature-responsive drug delivery as a critical research goal in localized cancer therapy.^13,14^

Conventional methods for treating malignant tumors include chemotherapy, radiotherapy, and surgical intervention.^15,16^ These methods of treatment have major drawbacks such as indiscriminate drug distribution, toxicity throughout the body, resistance to multiple drugs, and low availability of chemotherapeutic agents. Conventional chemotherapy includes the use of cytotoxic drugs that can negatively impact both cancerous and healthy tissues, causing harsh side effects with reduced efficiency in treatment.^17,18^ Nanomaterial-based drug delivery systems have appeared as platforms that hold promise for solving these issues by enhancing the targeted delivery of therapeutic agents. These nanoscale carriers offer improved drug encapsulation, controlled release behavior, and accumulation at tumor sites *via* passive or stimuli-responsive mechanisms to be able to increase therapeutic efficacy while reducing unwanted side effects.

Smart drug delivery schemes are those that reply to physiological triggers. The pH and temperature sensitivity of the DOX@MOF membrane has been evaluated in detail under simulated tumor microenvironment conditions. Drug release experiments revealed a significantly higher release rate for DOX at acidic pH (pH 5.0), which represents the extracellular pH of most solid tumors, compared to more neutral physiological pHs (6.2 and 7.4). Furthermore, drug release was enhanced considerably at higher temperatures, particularly at 37 °C, thus proving the dual-responsiveness of this system. This indicates that such a membrane would be highly suitable for targeted drug delivery so that drugs can be released only at diseased tissues with less effect on nearby healthy cells.^17,18^

The DOX@MOF nanofiber membrane showed more therapeutic properties apart from its use in drug delivery. *In vitro* studies revealed high cytotoxicity against different cancer cell lines, which highlights the potential use of the membrane for cancer therapy. Its antioxidant property was tested through radical-scavenging assays, while antimicrobial activity tests confirmed its effectiveness against key pathogenic microorganisms like *Escherichia coli*, *Staphylococcus aureus*, and *Candida albicans*. Such diverse biological activities indicate that this nanofiber membrane can be applied not only as a controlled drug delivery scheme but also for enhancing wound healing and infection control and lowering oxidative stress in medical applications.^19^

To enhance the reproducibility and efficiency of the formulated system, a Box-Behnken design was utilized as a statistical experimental approach for the optimization of formulations.^9,20^ With this approach, we were able to comprehensively evaluate the influence of various design variables on the characteristics of drug release. Optimization studies have successfully identified the optimal settings for achieving maximum drug loading, sustained release properties, and enhanced biological activity. The application of statistical design methodologies in this study not only adds scientific rigor to the manuscript but also lays the groundwork for future scale-up production that is reproducible and economically viable.

The innovation of this work deceits in the construction of a multipurpose drug delivery system that combines the advantages of V-MOFs, CS/PVA electrospun nanofibers, and DOX for chemotherapy application. The DOX@V-MOF membrane offers not only targeted and stimuli-responsive drug release but also possesses other functionalities such as antibacterial, antioxidant, and anticancer properties. Comprehensive physicochemical characterization and statistical optimization validate claims on the prospective biomedical applicability of this system specifically concerning cancer therapy. This study thus represents an immense contribution to a rapidly advancing field like nanomedicine while providing foundational support toward developing next-generation drug delivery systems tailored for precision medicine strategies.

## 2. Experimental

### 2.1. Instruments and materials

The substances used in this work, as shown in Table S1, were of analytical quality and were used as established without further purification. The instruments used in the study are described in detail in Table S2.

### 2.2. Drug carrier synthesis

#### 2.2.1. V-MOF synthesis

**Fig. 1:**
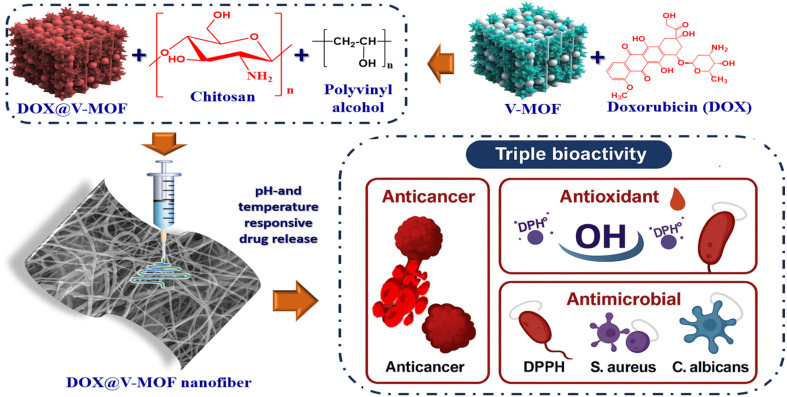
Schematic diagram of loading DOX onto V-MOF and its biological application.

The assembly of V-MOF at ambient temperature was approved out in alignment with the standardized methodologies described in prior research.^5^ In the course of this experiment, 2.38 mmol of benzene-1,3,5-tricarboxylic acid (0.50 g) was dissolved in 15 mL of a solvent system made up of equal parts ethanol (EtOH), dimethylformamide (DMF), and water. At the same time, 4.34 mmol of vanadium chloride (0.682 g) was also dissolved in 15 mL of the identical mixture of solvents.^5^ To create a homogenous solution, the two solutions were combined while being continuously stirred. After that, this mixture was stirred with 0.5 mL of triethylamine for 10 hours. After the stirring was done, the mixture was treated hydrothermally at a constant temperature of 80 °C for six hours. Following the heating process, centrifugation was used to separate the solid, and 20 mL of DMF were used to completely wash it.^21–23^ The substance was then dried for an additional six hours at 75 °C (Fig. 1). Hydrothermal synthesis of MOFs is mostly done at elevated temperature. However, the V-MOF used in this study was made at 80 °C under controlled solvothermal conditions to maintain the structural integrity and avoid any possible degradation of the framework during later steps for drug loading. The crystallinity of the manufactured material was confirmed by distinct diffraction peaks in the XRD pattern that meant an ordered framework structure had been formed. Though not specifically optimized, a synthesis yield produced a V-MOF with sufficient structural stability to be incorporated into a nanofiber membrane for use in delivering drugs afterward.

#### 2.2.2. Process for drug loading (DOX@V-MOF)

A proposed internal synthesis method shows that DOX cannot get into V-MOF because both doxorubicin and V-MOF keep a positive charge under the given experimental conditions. This means that post-synthesis of V-MOF needs a more controlled environment for the successful insertion of doxorubicin. We support an easy but effectual method for the insertion of drugs inside V-MOF.^24^ V-MOF (0.10 g) was added to 2 mL of a 0.5 mM DOX solution, and the mixture was agitated for 48 hours. The product, referred to as drug-embedded V-MOF, was then centrifuged and washed multiple times with methanol before drying the sample for use in further experiments.^25^ The quantity of DOX loaded into the V-MOF was indirectly measured using UV-visible spectrophotometry. A DOX calibration curve covering the concentration range of 1–20 mg L^−1^ was created in buffer solution of phosphate (pH 7.4). Each standard solution was read at its maximum absorption wavelength (*λ*_max_ = 480 nm), which corresponds to the characteristic π–π* transition of DOX. The plot of absorbance *vs.* concentration showed good linearity (*R*^2^ > 0.99). The supernatant after centrifugation post drug loading was tested under the same conditions to find out how much DOX had not bound. Drug loading content and efficiency were calculated from the change among initial and final concentrations of DOX in the supernatant solution. This method has several significant benefits compared to conventional drug encapsulation approaches described in eqn (1) and (2):1
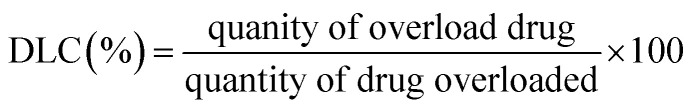
2
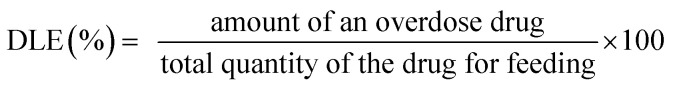


#### 2.2.3. Creation of a DOX@V-MOF nanofiber membrane

Nanofiber scaffolds were produced using electrospinning technology. During this study, a polyvinyl alcohol (PVA) solution at 15% weight/volume (w/v) in 90% acetic acid was organized. This PVA solution was mixed with chitosan (CS) at 2% (w/v) concentration in 90% acetic acid medium, at an exact volumetric ratio of 7 : 3 for both components. The next step in the protocol involved adding DOX-MOFs to the resulting solution. It was agitated for one night to create a uniform suspension. Following this homogenization procedure, a 20-gauge blunt-tip needle was used to move the solution of polymers through a glass syringe.^26^ The solution was delivered at a regulated flow rate of 1 mL h^−1^ using a syringe pump.^27^ A high-voltage power source was employed to deliver a voltage between 27 and 28.5 kV at the needle, that was positioned 15 cm above a grounded collection plate, in order to enhance the electrospinning processes (Fig. 1).

#### 2.2.4. *In vitro* DOX release study at different pH conditions

The investigation of DOX's *in vitro* release behavior from the nanofiber membrane that contained DOX@V-MOF was performed under various pH conditions to mimic physiological conditions and those found in tumor microenvironments.^28^ To put it briefly, 25 mL of buffer solutions at pH 7.4 and 6.2 (phosphate buffer) and pH 5.0 (acetate buffer) were mixed with 10 mg of the DOX@V-MOF membrane.^29^ The experiments for release took place at 37 °C with constant agitation at 150 rpm. At set times, 1 mL of the release medium was taken out and centrifuged to clarify any particles. The amount of DOX that had been released into the effluent was quantified by UV-visible spectrophotometry using an already created calibration curve. To maintain a consistent volume, an equal volume of new buffer solution was introduced after each sampling.^17^ The findings are shown as mean values with matching standard deviations for each drug release investigation, which was conducted in triplicate. Using eqn (3), the overall percentage of DOX generated was determined.3
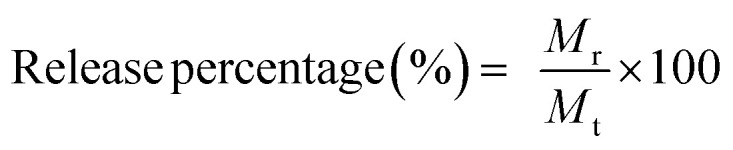
In this case, *M*_t_ denotes the total quantity of DOX that has been integrated, while *M*_r_ denotes the amount of DOX that has been released.

### 2.3. Activities related to biology

#### 2.3.1. Activity of antioxidants

The ability of V-MOF membrane, free DOX, and DOX@V-MOF nanofiber membranes to scavenge radicals was assessed using the DPPH test technique. The DPPH radical, also known as 2,2-diphenyl-1-picrylhydrazyl, was made and kept at 10 °C in the dark. A concentration of 0.004 percent (w/v) of the radical disappeared in newly made methanol. Following this procedure, all test tasters were organized in a methanol solvent. For testing purposes, 40 µL from each test sample was added into 3 mL of the DPPH solution for measurement.^30^ A prompt test of absorbance standards was done with a UV-visible spectrophotometry. At 515 nm, the absorbance kept going down steadily, with readings taken every min until it stabilized. This was seen at the 15-min mark. Also, the control DPPH radical absorbance and that of the ascorbic acid (standard compound) were checked. After three repetitions of each test condition, the data were averaged for additional analysis.^31^ The following eqn (4) was used to determine the DPPH radical's percentage of inhibition (PI):4
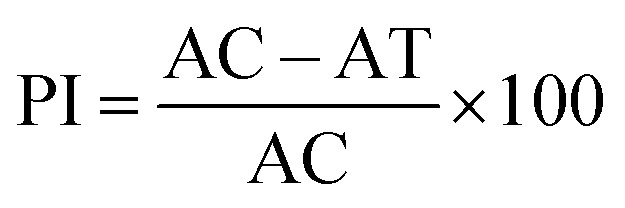
In this instance, AC is the control's absorbance measurement at time *t* = 0 min. Conversely, at time point *t* = 16 min, AT is the absorbance of a sample that has interacted with DPPH.

#### 2.3.2. Cytotoxicity evaluation using the viability assay

The MCF-7 human breast cancer cell line and the HepG-2 human hepatocellular carcinoma cell line were obtained from the American Type Culture Collection (ATCC) in Rockville, Maryland, and were utilized in this investigation. To preserve their viability, these cultures were kept in Dulbecco's Modified Eagle's Medium (DMEM) supplemented with 10% heat-inactivated fetal bovine serum, 1% l-glutamine, and gentamicin at a concentration of 50 µg mL^−1^. Every two weeks, cells were subcultured in an incubator set at 37 °C with a humidified environment that contained 5% carbon dioxide.^32^

In order to perform the cytotoxicity test, 100 µL of growth media was applied to a 96-well plate containing 10 000 cells per well. Fresh medium containing different concentrations of the test chemical were added after a 24-hour incubation period. A multichannel pipette was used to precisely deliver the test substance into flat-bottomed microtiter plates (Falcon, NJ, USA) after it had been serially diluted twice and applied to confluent cell layers. These plates were then incubated at 37 °C with 5% CO_2_ for a full day. The test compound was added to three wells per concentration. Control cells were kept separate from treatment wells; there was no need to add dimethyl sulfoxide (DMSO) since it did not affect the results when included up to a maximum concentration of 0.1 percent in the wells. At the end of an additional 24-hour incubation at 37 °C, viable cell counts were determined by colorimetric assay.^33^

In conclusion, the liquid was extracted from each individual well at the conclusion of the incubation period. After that, a 1% crystal violet solution was applied to these wells for a minimum of half an hour. Following staining, plates were rinsed with distilled water to remove any remaining stain and color. After that, 30% glacial acetic acid was then added to each well and thoroughly mixed. A microplate reader (TECAN, Inc.) was used to measure the plates' absorbance at 490 nm. To standardize the results, absorbance measurements from wells that were not stained further were used as background controls. As a control, cells that were not exposed to the investigated chemicals were contrasted with treated samples. All experiments were done in duplicates for reliability of results as shown in eqn (5). Each compound's cytotoxicity was evaluated, and cell viability was assessed using a microplate reader to measure optical density (eqn (5)).^34^5
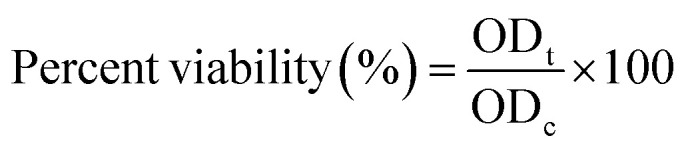


The optical density for wells treated with the particular sample under test is denoted by OD_t_, while the optical density for control wells containing untreated cells is denoted by OD_c_. To ascertain the impact of the designated chemical on each tumor cell line, mortality curves were created by charting cell survival *versus* the concentration of the chemical. Graph Pad Prism was used to calculate the 50% inhibitory concentration (IC_50_). The IC_50_ values were based upon graphical outputs from dose–response curves for each concentration of tested chemical.^32^

#### 2.3.3. Antimicrobial research

*Escherichia coli* (*E. coli* ATCC 25955), a Gram-negative bacterium, was cultivated in newly made growth media for this investigation. Simultaneously, the antibacterial activity of V-MOF nanofiber membranes was tested using *Staphylococcus aureus* (*S. aureus* ATCC 25923), a Gram-positive bacterium. Additionally, DOX@V-MOF and free DOX nanofiber membranes were evaluated.^35^ The agar-well diffusion method was used to conduct the evaluation. Initially, each sterile Petri dish was filled with 40 mL of LB Agar media containing the designated bacterial strains. After that, the dishes were left to solidify at room temperature.^36^ In current study, wells of 6 mm diameter were made using sterilized cork borer. The culture plates used in this study were incubated for 24 hours at 37 °C. One of three treatment groups, V-MOF nanofiber, free DOX, or DOX-loaded V-MOF nanofiber membrane produced at a concentration of 10 mg mL^−1^ was added to each well in 100 µL. To determine the antibacterial activity of the materials being studied, the widths of the inhibition zones that developed after the incubation period were evaluated. These inhibitory zones were then contrasted with those created by the common antibiotic Gentamicin against strains of both Gram-positive and Gram-negative bacteria.

### 2.4. Drug delivery system based on pH

A widely studied method for accurately delivering drugs to cancer places includes the use of biomaterials that are responsive to the changes in pH typical of the tumor microenvironment.^37^ The pH differential between surrounding healthy tissues and tumor microenvironments has been cleverly used to create a pH-responsive medication delivery device. The development of therapeutic medicines with enhanced responsiveness is made possible by this fluctuation in pH gradients. Materials in these settings may undergo structural changes as a result of pH shifts from neutral to acidic. Pharmaceutical delivery systems that use V-MOFs, which expand in response to pH changes and aid in the release of medicinal substances, stand out among them. The fundamental principles of these pH-sensitive drug delivery techniques often entail the protonation or deprotonation of acidic or amine attached groups, which result in volumetric alterations as a result of variations in external pH conditions.^35^

### 2.5. Design of experiments

A sophisticated statistical method called response surface methodology (RSM) is used to fit complicated models in scenarios with several alternative outcomes. This method's primary objective is to explain how the variable that responds and several experimental factors relate to one another.^38^ Additionally, applying these models enhances the processes under investigation's fine-tuning. The sorption parameters of each tested material were determined by averaging the outcomes of three distinct experimental trials. Finding the optimal outcome within the entire methodology is made easier by RSM's methodical approach, which consists of a number of planned tests intended to identify the most crucial process factors. A frequently used method for optimizing procedure parameters is the central composite design (CCD).^39^ Three main variables that control the optimization procedure are “temperature”, “interaction time” and “solution pH”. An assessment revealed that these variables have a significant negative effect on adsorption capacity, as indicated by information in Table S3.

Extreme values of all parameters tested are given in Table S3. These came from a thorough examination that was done with Design Expert software. This table displays the results for the different borders and their corresponding outcomes. Center runs (*P*), axial runs (2 × *m*), factorial runs (2^*m*^), and other design strategies and mixes utilized in the study are all methodically categorized in the discussion.^40^ This set of approaches highlights the complex characteristics of the employed research methods. The formula given here serves as a framework to determine how many experimental trials are needed based on the number of input variables involved in eqn (6).

6 *N*_p_ = [2^*m*^ + (2 × *m*) + *P*] = [2^3^ + (2 × 3) + 3] = 17

*P* is the total number of significant research experiments carried out, and *N* is the number of practical variables that affect the outcomes. In this study, we assign a value of “*m*” equal to 3. Assessing the model's coefficients, planning the experiment, and forecasting the model's results are the three primary components of the central composite design. A thorough and in-depth analysis of the outcomes at each of these stages is required.^40^ After completing the tasks described above, an empirical model has been created to analyze the performance of the function under different scenarios with varying input variables. As a result, eqn (7) illustrates the fitting of a quadratic regression model:

7 *Y* = *β*_0_ + ∑ *β*_*i*_*X*_*i*_ + ∑ *β*_*ii*_*X*_*i*_^2^ + ∑∑*β*_*ij*_*X*_*i*_*X*_*j*_

The velocity coefficient is represented by “*j*” and the resistance constant by “*I*” in this analysis. The constants *β*_0_, *β*_*i*_, *β*_*ii*_, and *β*_*ij*_ correspond to the constant term, resistance, interaction, and velocity, respectively. As indicated in Table S4, the evaluation metrics *R*^2^, *R*^2^_Adj_, and *R*^2^_Pred_ were used to assess how well the suggested polynomial equation fits the data. A large *R*^2^ value shows an enhancement in the model's predictive validity indicating stronger correspondence among the model and experimental data.^40^

## 3. Results and discussion

### 3.1. Description of DOX@V-MOF nanofiber membrane

#### 3.1.1. X-ray diffraction (XRD) patterns

**Fig. 2:**
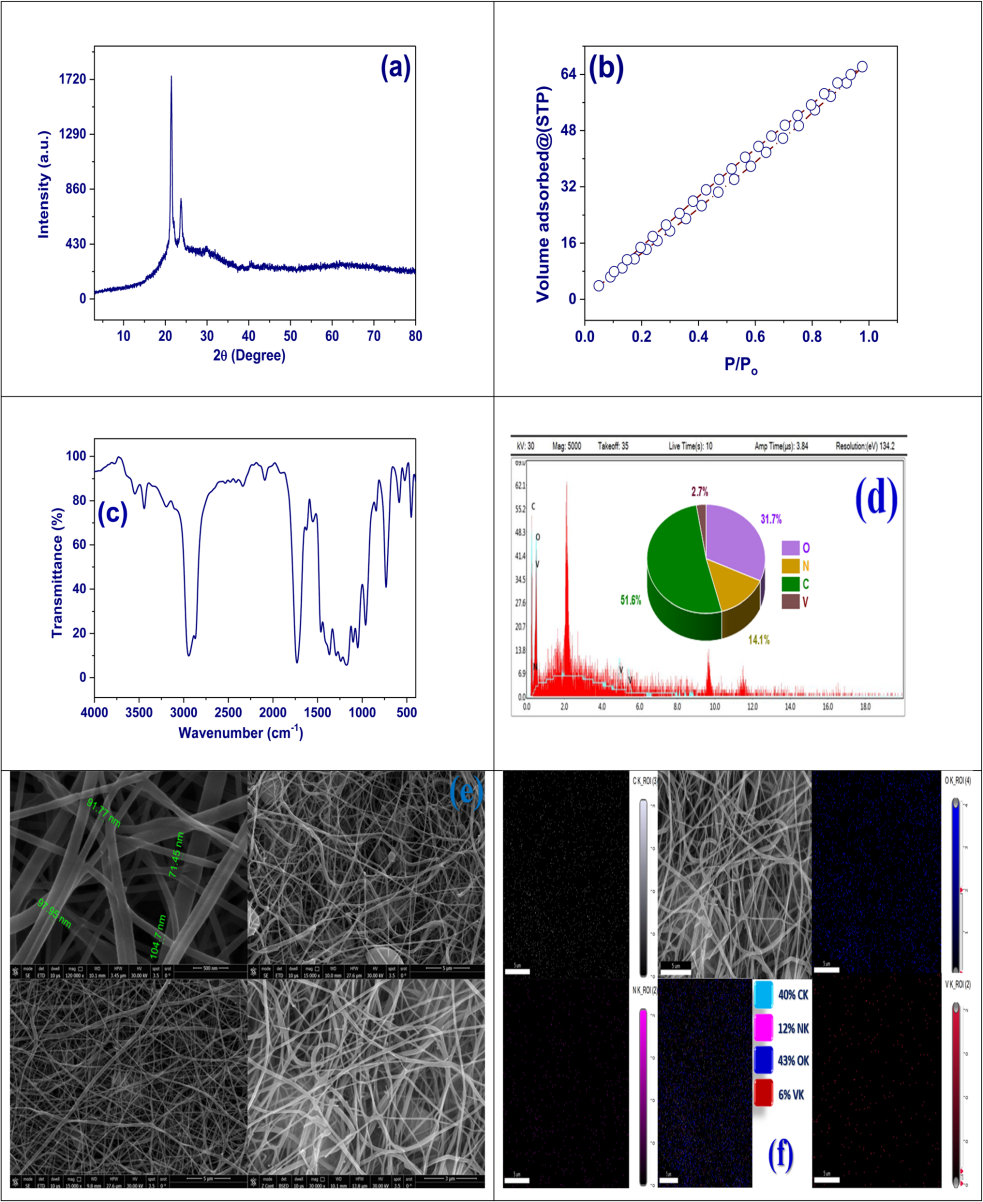
(a) The DOX@V-MOF nanofiber membrane's XRD pattern, (b) isotherm of N_2_ adsorption/desorption of DOX@V-MOF nanofiber membrane, (c) DOX@V-MOF nanofiber membrane FT-IR, (d) DOX@V-MOF nanofiber membrane EDX analysis, (e) SEM image at different scale, and (f) DOX@V-MOF nanofiber membrane SEM mapping.

X-ray diffraction patterns for the DOX@V-MOF nanofiber composite show strong and well-defined peaks mostly in the 2*θ* variety of 10° to 30°. The most intense diffraction peak at about 21° indicates a highly ordered and crystalline structure typical for V-MOFs. Also, another peak that appears around 25° confirms that there is a long-range periodicity continuing in the lattice structure of the MOF.^41^ The peaks show a very clear and strong intensity, which means that the crystalline assembly is mostly conserved after the DOX is added. There are no new peaks or fuzzy halos that would usually show structural damage or major disorder. The lack of any new diffraction patterns means that DOX got in through physical trapping and not chemical change; thus, it kept the original crystal structure. Maintaining the structural integrity is critical for preserving the required porosity and stability for controlled drug release. XRD analysis in Fig. 2(a) illustrates how crystallinity remains stable and compatible with biological applications for the system DOX@V-MOF, as well as its effective formulation.

#### 3.1.2. Isotherm of N_2_ adsorption/desorption

The isotherm of N_2_ adsorption/desorption from the DOX@V-MOF nanofiber membrane shows Type IV isotherm behavior, which signifies mesoporous constituents. It indicates a gradual increase in nitrogen adsorption over the relative pressure range (*P*/*P*_0_), with a nearly linear segment observed between 0.05 and 0.3. The BET surface area calculated from this data is 112.52 m^2^ g^−1^, demonstrating the attendance of a highly porous assembly that favors applications pertaining to adsorption and molecular loading such as drug delivery.^41^ The total pore volume at a relative pressure of *P*/*P*_0_ ≈ 0.99 is 0.12 cm^3^ g^−1^, indicating the presence of accessible mesopores in the material. It can be expected from the isotherm image, which shows only the adsorption branch, that a Type H3 hysteresis loop will appear during desorption. This type of hysteresis usually results from the formation of non-rigid collections with plate-like subdivisions or slit-shaped pore systems. The observed hysteresis means that capillary condensation happens in the mesopores and somewhat describes the irreversibility related to nitrogen desorption.^42^ This indicates that the DOX@V-MOF system has a high encapsulation efficiency and a sustained release profile. In brief, the isotherm results, in combination with the surface area and pore characteristics, highlight the structural integrity of the nanofiber membrane. As such, it is highly suitable for biomedical applications requiring precise control of molecular interactions, as exemplified in Fig. 2(b).

#### 3.1.3. FT-IR

The DOX@V-MOF nanofiber membrane's FTIR spectrum is shown in Fig. 2(c). Identifying the bonding contacts and functional groups found in the composite structure requires knowledge of this spectrum. The existence of hydroxyl and amine groups commonly associated with CS/PVA and the DOX drug molecule is shown by the broad absorption band above 3000 cm^−1^.^43^ The peaks found around 2900 cm^−1^ are related to aliphatic chains' C–H stretching, which is a feature of the polymer core of the nanofiber matrix. Between 1720 and 1650 cm^−1^, a major absorption band appears due to C=O bond stretching vibrations from quinone and ketone functional groups in the DOX molecule. The region from 1600 to 1500 cm^−1^ displays C=C stretching vibrations of an aromatic nature, indicating that the typical aromatic structure of DOX has been retained. Also, new bands in the variety of 1450–1300 cm^−1^ can be assigned to C–N stretching and bending vibrations; hence, this confirms more clearly the presence of DOX.^44^ On the other hand, in the low wavenumber region, between 700 and 500 cm^−1^, strong absorption bands due to V–O stretching vibrations of the structural framework of the V-MOF are detected. These bands are practically identical to those found in the parent material, and the presence of these specific signals with minimal shifts in intensity or position is indicative of possible physical interactions. C=O stretching vibrations due to quinone and ketone functional groups from the DOX molecule produce a strong absorption band between 1720–1650 cm^−1^ without disrupting its crystalline structure; this serves as further proof that DOX has been successfully loaded into MOF matrixes and highlights its potential request in drug delivery schemes.

#### 3.1.4. EDX analysis

Fig. 2(d) shows the energy-dispersive X-ray spectrum besides elemental analysis map of the DOX@V-MOF nanofiber membrane, which provide explicit details regarding the chemical composition examination of the synthesized resources. The EDX spectrum exhibits distinct peaks for carbon, nitrogen, oxygen, and vanadium elements that confirm successful loading of drug molecules as well as V-MOFs into polymeric nanofibers.^45^ The pie chart gives information about the atomic percentages of the elements. It can be seen from the chart that carbon has the largest percentage, which is 51.6%. This can be explained by the fact that it comes from both the polymer backbone and organic frameworks contained in DOX and V-MOF. The next element found in greater amounts is oxygen with an amount equal to 31.7%, which may indicate a large number of oxygen-containing functional groups in both the structure of MOF and that of the molecule DOX. Nitrogen makes up 14.1%, which would mean there are some nitrogen-rich useful groups; these could be amines coming from DOX or possibly those connected with ligands in the MOF.^46^ Presence of vanadium 2.7% proves successful introduction of V-MOF into nanofibers. This elemental makeup matches the intended design for the DOX@V-MOF composite and confirms its functional operation in biomedical applications especially concerning metal participation and drug efficacy as shown in Fig. 2(d).

#### 3.1.5. SEM analysis

The prominent peaks observed in the energy-dispersive X-ray (EDX) spectrum, corresponding to the elements C, N, O, and V, confirm the effective loading of DOX and V-MOFs into the polymeric nanofiber matrix, thereby validating the successful implementation of the electrospinning technique for fabrication. Additionally, at higher magnifications, individual nanofibers exhibit smooth surfaces with diameters ranging from approximately 71.45 to 104.7 nm, indicating well-controlled fabrication parameters and reflecting an exceptional case of bead formation or morphological defects.^47^ To enhance drug loading and regulator the release mechanism, the fibers should have a dense and uniformly spread morphology. This is crucial for increasing surface area and promoting porosity. The images taken at lower magnifications (top right and bottom panels) provide a closer view of the fibrous membrane which indicates that a uniform three-dimensional network has developed with fibers interlaced; thus, adding mechanical strength to allow molecular diffusion. The addition of V-MOF appears to retain the general shape suggesting good compatibility as well as dispersal of V-MOF inside the polymer matrix. In summary, scanning electron microscopy (SEM) analysis has confirmed that DOX@V-MOF nanofibers possess appropriate morphological characteristics necessary for biomedical applications particularly concerning drug delivery. Such properties significantly influence performance such as surface area, porosity, and structural stability as depicted in Fig. 2(e).

The elemental mapping image from scanning electron microscopy-energy dispersive spectroscopy (SEM-EDS) shows that the structure of the DOX@V-MOF nanofiber membrane has an architecture of continuously ordered and interconnected fibers. The primary SEM micrograph indicates that nanofibers are arranged in a highly porous, well-distributed network that would be very favorable for drug loading besides release applications.^48^ The uniform distribution of carbon confirms the organic nature of both the polymeric and pharmaceutical components. Nitrogen, associated with the amine groups in chitosan and nitrogen-rich ligands within the V-MOF, also shows an even spread throughout the composite matrix. Oxygen is a major constituent from both DOX and the MOF setup. It displays a dense and homogenous distribution that indicates an oxygen-rich characteristic for this nanocomposite. Although vanadium has a relatively low concentration of 6%, its uniform distribution confirms successful incorporation of the vanadium-based MOF into the fibrous structure.^49^ The elemental composition and percentages in the color-coded legend are 40% carbon, 12% nitrogen, 43% oxygen, and 6% vanadium. This further validates the successful fabrication of a composite nanofiber membrane with evenly distributed functional materials, as described in Fig. 2(f).

#### 3.1.6. XPS study

**Fig. 3:**
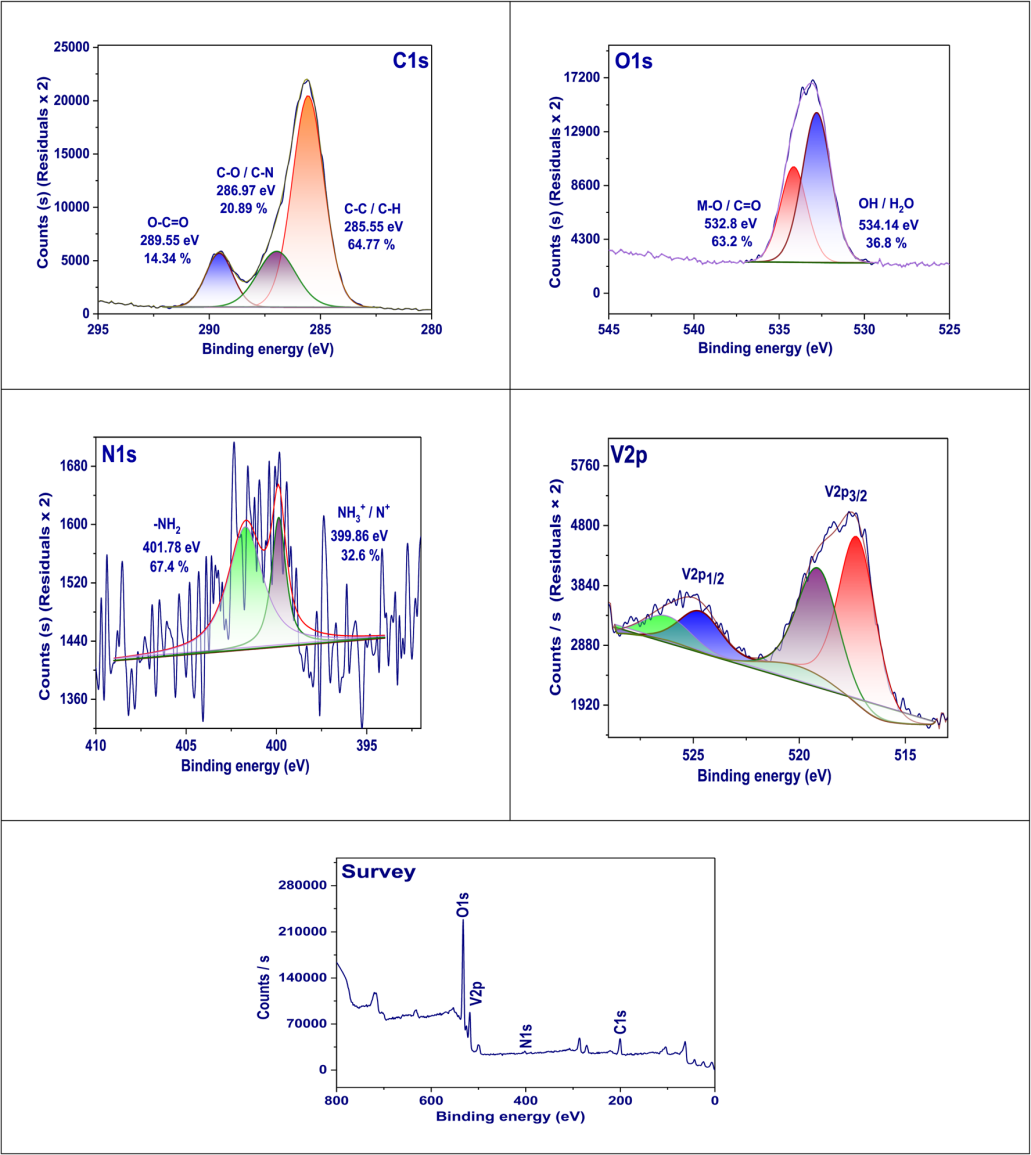
XPS pattern of the nanofiber membrane DOX@V-MOF.

The high-resolution X-ray photoelectron spectroscopy (XPS) study of the C 1s region pertaining to the DOX@V-MOF nanofiber membrane reveals intimate details about chemical environments and bonding properties associated with carbon atoms within this composite material.^50^ The spectrum that has been separated shows three major peaks at binding energies of 285.55, 286.97, and 289.55 eV; these are associated with various carbon functional groups. The peak at 285.55 eV is most important because it makes up 64.77% of the total carbon signal and is connected with C–C/C–H bonds that are usually found in the aliphatic backbone of the polymer matrix and also in the hydrophobic parts of DOX. The next peak at 286.97 eV makes up 20.89% of the total intensity and indicates C–O/C–N bonds; this means hydroxyl, ether, and amine groups from both the DOX molecule and organic ligands from the MOF are present. The third peak, which appears at 289.55 eV and contributes 14.34% to the overall carbon signal, is related to O–C=O (carboxyl) functional groups probably coming from surface-bound carboxylic acids or ester functionalities. These useful groups play an important role in increasing hydrophilicity enabling drug interaction as well as coordination with metal centers as shown in Fig. 3.

O 1s region XPS examination on the DOX@V-MOF membrane brings out important information regarding the various biochemical states of oxygen existing in this compound material. The O 1s spectrum analysis shows two main peaks at 532.8 and 534.14 eV, which suggest different possible conditions for oxygen. The bigger peak at 532.8 eV, which accounts for 63.2% of the total O 1s signal, is credited to lattice oxygen (V–O bonds) or C=O groups that are usually found in the framework oxide of V-MOF and carbonyl groups from DOX itself. The smaller secondary peak at 534.14 eV, which constitutes about 36.8% of the signal, is associated with hydroxyl (–OH) or physically adsorbed H_2_O molecules.^51^ It can be seen from the above that the composite is hydrophilic and contains oxygen species on its surface. The oxygen functionalities identified here play a significant role in defining the physicochemical properties of a material, for example, metal coordination, hydrogen bonding, and interactions with biological targets (Fig. 3).

The N 1s high-resolution XPS spectrum of the DOX@V-MOF nanofiber membrane is critical for elucidating the chemical states of nitrogen, which are essential for understanding the fully biological and coordination properties of the material. In addition, O 1s XPS results confirm that chemically active oxygen-containing functional groups have been successfully introduced into the DOX@V-MOF nanofibers to enhance their bioactivity, drug binding capability, and therapeutic efficiency.^52^ The main peak at 401.78 eV, which is 67.4% of the total N 1s signal, is assigned to protonated amine or quaternary nitrogen species (–NH_3_^+^ or N^+^). These are usually found in DOX molecules and could also be due to nitrogen from within the metal–organic framework, suggesting strong bonding between the nitrogen atoms and nearby structural parts or possibly surface protonation. The other peak at 399.86 eV, making up 32.6% of the signal, belongs to imine-type nitrogen or neutral amine functionalities (–NH_2_), indicating that there are unbound nitrogen species from both the DOX and MOF ligand systems. Different states of nitrogen play an important role in increasing electrostatic interaction, hydrogen bonding, and possible coordination with metals for more biological activity of the material as well as better drug delivery properties. Therefore, the N 1s XPS profile confirms that nitrogen-rich functionalities have been successfully introduced and retained in the DOX@V-MOF nanocomposite.

The oxidation states and chemical surroundings of vanadium in the composite material are thoroughly examined through the high-resolution XPS spectrum in the V 2p region for the DOX@V-MOF nanofiber membrane. The V 2p spectra show two separate components of the spin–orbit doublet, which correspond to V 2p_3/2_ and V 2p_1/2_ levels; hence, they represent different species of vanadium.^53^ The V 2p_3/2_ peak appears at lower binding energies (about 516–518 eV), while V 2p_1/2_ is found at higher energies (about 523–525 eV). This is in line with the expected results for vanadium in its common oxidation states. A more detailed examination of these peaks shows several oxidation states that are usually associated with V^4+^ and V^5+^ ions. This indicates that there is a mixed valence condition in the V-MOF. Mixed valency plays critical roles in drug delivery and catalysis by enhancing redox activity and overall functionality. The difference in peak intensities as well as their separation further suggests that vanadium has been strongly bonded into the nanofiber matrix and holds onto its electronic configuration within the composite structure.

The XPS survey spectrum of the DOX@V-MOF membrane offers a comprehensive view of the surface elemental composition of the sample. The peaks conforming to C 1s, N 1s, O 1s, and V 2p are clearly visible in this spectrum, indicating that both organic and inorganic components have been successfully integrated into the composite material.^54^ The carbon atoms in the polymeric matrix and the DOX molecules, which acted as organic ligands in the MOF, correspond to the C 1s peak detected at about 285 eV. The attendance of useful groups containing nitrogen, such as amines and imines, from DOX and possibly from the MOF structure is indicated by an N 1s peak around 400 eV. An intense O 1s peak near 532 eV suggests that oxygen atoms are present in various environments, including carbonyl, hydroxyl, and metal–oxygen (V–O) configurations. A clearly defined V 2p peak within the range of 515–525 eV confirms the presence of vanadium species typical for V-MOF frameworks. No other or contaminant elements are seen in the spectrum further proving chemical purity of the created nanofiber membrane. This survey scan also acts as a primary confirmation for all major components involved in structure formation thus laying foundation for further detailed high resolution XPS studies on individual elements.^55^

XPS results revealed that vanadium happens in mixed oxidation states (V^4+^ and V^5+^) in the DOX@V-MOF nanofiber membrane, which may have a potential effect on its redox behavior. Such coexistence of oxidation states can facilitate electron transfer processes and thus contribute to the radical scavenging activity detected in the DPPH assay. However, the cytotoxic effects revealed by IC_50_ values result from the chemotherapeutic action of DOX and not from any intrinsic redox-mediated activities of vanadium species. It should also be noted that potential toxicity from vanadium-containing nanomaterials requires care, with further *in vivo* studies needed for long-term biosafety assessment and therapeutic applicability of such systems.

### 3.2. Controlled release of DOX drug

#### 3.2.1. Effect of pH

**Fig. 4:**
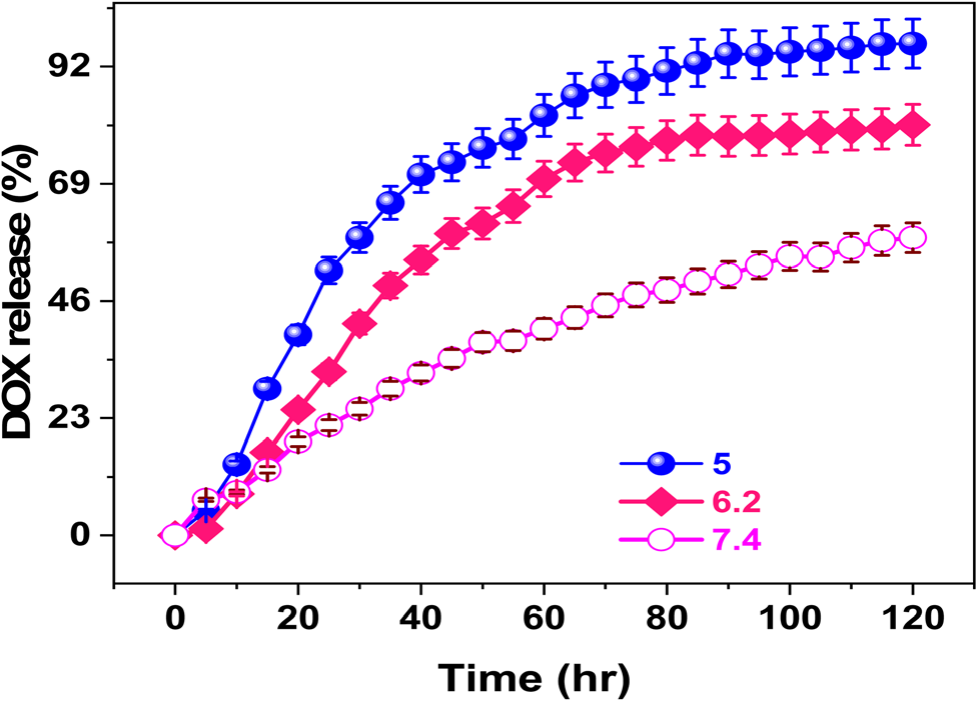
Cumulative percentage release of DOX from the DOX@V-MOF membrane at diverse pH values (5.0, 6.2, and 7.4) as a purpose of contact time at 37 °C. Error bars signify standard deviation (*n* = 3).

The drug loading capacity and encapsulation efficiency of the DOX-loaded V-MOF membrane was 87.8 and 96.6%, respectively. This confirms that the system is very effective at encapsulating DOX.^56^ To fully understand the release features, tests were done at three different pH levels: 7.4, 6.2, and 5.0. Careful control of the temperature at 25 °C for up to 120 hours was done. Results from these tests are shown in Fig. 4. The findings conclusively show that pH changes have a significant impact on the DOX releasing mechanism from the V-MOF nanofiber membrane. In particular, the release rate is significantly higher at pH 5.0 than at pH 7.4 and 6.2, indicating that the V-MOF nanofiber membrane becomes more sensitive to pH variations. The release rates of DOX from the V-MOF membrane were found to be 58.4, 80.6, and 96.5 at pH values of 7.4, 6.2, and 5.0 over a period of 140 hours, respectively. The kinetics suggest that the V-MOF membrane can greatly improve drug release near tumor places for better uptake by cancer cells. Therefore, this mechanism's pH-sensitive nature plays an important role in increasing DOX cytotoxicity and may improve treatment strategies (Fig. 4).

Hydrogen bonding contact of DOX with V-MOF membrane is a key factor in determining the kinetics of drug release. One important observation was that the amount of DOX released was meaningfully superior at pH 5.4 than at pH 7.4, which emphasizes its relevance since this pH value resembles the acidic nature of a tumor microenvironment; hence, it means that doxorubicin will be released more readily when conditions are similar to those found in tumors. Such a mechanism would be beneficial by possibly minimizing harmful effects on neighboring healthy tissues. The pH-dependent nature of the release mechanism can be explained by differences in the strength of hydrogen bonding interactions taking place between functional groups of DOX, particularly amino (–NH_2_) and hydroxyl (–OH) groups, with functional groups existing in the V-MOF membrane such as carboxylic (–COOH) and hydroxyl (–OH) groups.^56^ At a neutral pH, the enhanced hydrogen bonding interactions significantly contribute to reducing the release rate of DOX under these conditions.^57^ It has been reported that hydrogen bonding interactions are stronger in basic environments than in acidic environments. This difference in bonding strength plays a crucial role in the increased release of doxorubicin under acidic conditions. Once the drug-loaded nanocarrier is administered and guided to a tumor cell by an external magnetic field, it gets inside the tumor cell by endocytosis. The amine groups protonation presents in DOX will break the hydrogen bonding interactions among the V-MOF membrane and DOX, thus facilitating a faster rate of drug release. Additionally, more DOX is released at physiological temperatures compared to room temperature as shown in Fig. 4.

#### 3.2.2. Temperature effect

**Fig. 5:**
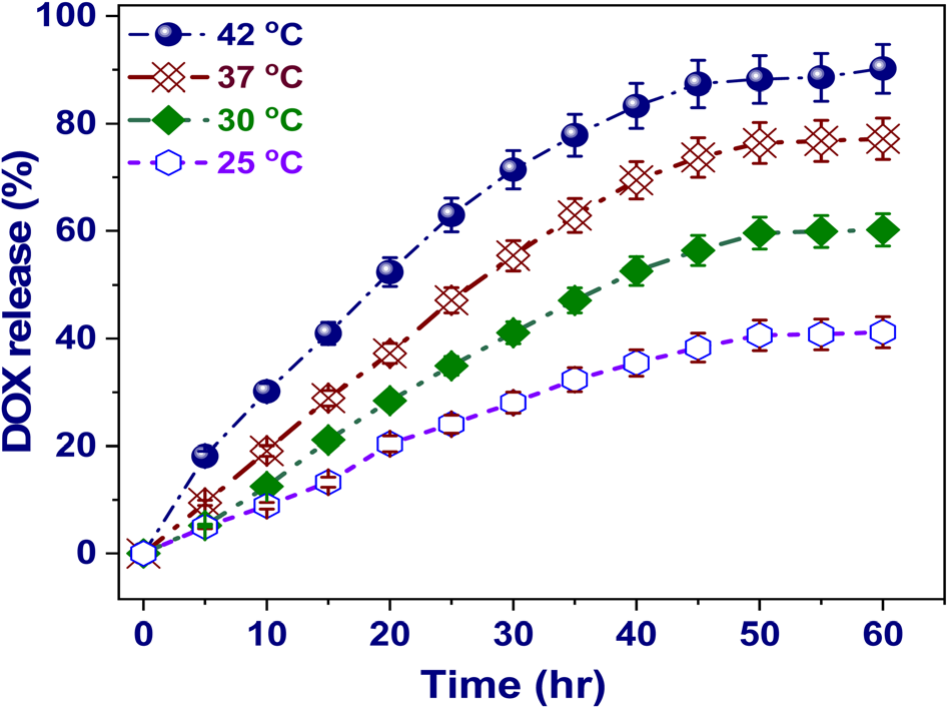
Effect of temperature (25–42 °C) on the cumulative release percentage of DOX from the DOX@V-MOF membrane as a function of contact time at pH 5.0. Error bars signify standard deviation (*n* = 3).

As illustrated in Fig. 5, the temperature influence on the release kinetics through the DOX@V-MOF nanofiber membranes were investigated throughout a 50-hour testing period at four distinct temperatures: 25, 30, 37, and 42 °C. Results indicated that there is a strong relationship between temperature and the release kinetics of DOX; with higher temperatures accelerating the process more quickly. The total release at 25 °C was about 41.2%, which meant that it was slow diffusion of DOX out of the nanofiber matrix. When the temperature increased to 30 °C first and then to 37 °C, there was a very big improvement in the efficiency of releases up to about 60.2 and 77.2%, respectively. It was observed that thermal conditions are very influential in altering the dynamics of drug release.^57^ A significant observation is made at a temperature of 42 °C, which simulates hyperthermic conditions usually found in tumor microenvironments. Here, total drug release surpasses 90.2%. This reinforces the thermally responsive characteristics of the nanofibers. The increase in drug release with temperature variation can be credited to some crucial factors. First, high temperatures increase molecular mobility and therefore help DOX to diffuse through the porous DOX@V-MOF membrane. It is also expected that both pore size and permeability will increase due to thermal expansion of the nanofiber matrix. In addition, interactions like hydrogen bonding and coordination between DOX and functional groups inside V-MOF structure especially hydroxyl (–OH) and carboxyl (–COOH) groups are reduced by thermal effects; this reduction in binding affinity favors drug desorption. The temperature-responsive release mechanisms have great advantages for cancer therapy because they permit localized hyperthermia to selectively release drugs at specific sites, hence reducing systemic side effects while improving treatment efficacy. This finding underscores the potential of the DOX@V-MOF membrane as a state-of-the-art drug delivery platform with thermal responsiveness and controlled capability for precise administration of chemotherapy.^58^

### 3.3. Kinetic models

**Fig. 6:**
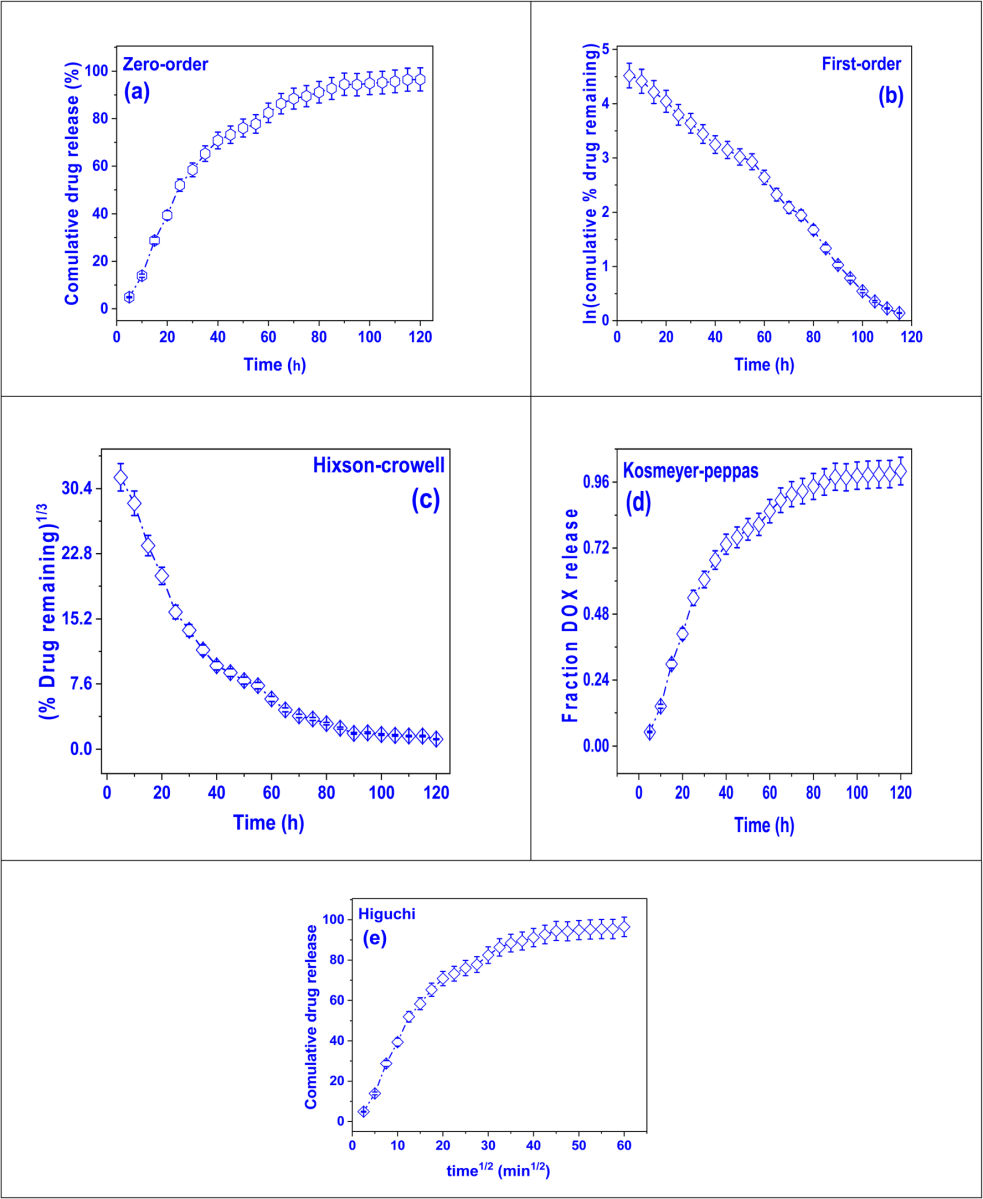
Drug release kinetic models for DOX release from DOX@V-MOF membrane: (a) zero-order model, (b) first-order model, (c) Hixson-Crowell model, (d) Korsmeyer-Peppas model, and (e) Higuchi model. Error bars signify standard deviation (*n* = 3).

The model of zero-order kinetic, which assumes that the release rate is independent of drug concentration, was used to study the release behavior of DOX from DOX@V-MOF. The increasing percentage of drug released increased linearly with time in the profile presented in Fig. 6(a) and reached about 90–95% after 120 hours. This linear trend is indicative of sustained and controlled release behavior following zero-order kinetics.^59,60^ The model fits the experimental data very well using a well-defined zero-order release rate constant (*K*_0_) of 1.12 h^−1^.^60^ The sustained release profile of the DOX@V-MOF membrane indicates zero-order kinetics, which minimizes the burst release effect and maintains a prolonged therapeutic concentration. Such controlled release behavior is highly favorable for anticancer therapy, where achieving and sustaining a high local drug concentration is crucial for maximizing therapeutic efficacy while minimizing systemic toxicity. Therefore, the kinetic studies provide strong support for the assertion that the DOX@V-MOF system exhibits sustained release behavior, as detailed in Table S5.

The model of first-order kinetic was used to evaluate the release behavior of DOX from the DOX@V-MOF nanocomposite. According to this model, the quantity of drug that remains in the delivery scheme is directly proportional to the rate of its release. A plot of ln (cumulative percentage of residual drug) *versus* time shows a straight line, which confirms that the first-order model is applicable. This relationship can be seen in the release profile presented in Fig. 6(b).^61^ The linearity detected indicates that the release rate of the drug decreases exponentially with time, which is common in systems where the release device is administrated by concentration-dependent diffusion. The first-order release rate constant (*K*_F_) was determined to be 0.39 h^−1^, indicating a moderate release rate that decreases with decreasing drug concentration; such kinetic behavior would suggest that the DOX@V-MOF system would provide initially a relatively high release rate which then falls off – such a profile could be desirable in applications where an initial therapeutic burst is needed followed by maintenance at lower levels. First-order kinetics, rather than zero-order release, would result in less stable drug concentrations over time but provide very valuable insight into the actual release mechanisms and also help to understand the extent of the concentration effect on diffusion-controlled release from MOF matrices (Table S6).

The model of Hixson-Crowell kinetic was used to further understand the device of drug release of DOX from the DOX@V-MOF nanocomposite.^62^ In-depth studies were conducted on how variations in surface area and particle size affect the dissolution profile. From the plot shown in Fig. 6(c), cube root of fraction drug remaining *versus* time has been reported as a nonlinear profile. This demonstrates that the drug release mechanism is measured by the slow erosion or disintegration of the matrix containing the drug. The Hixson-Crowell release rate constant, *k*_HC_, was found to be 0.77 h^−1^ indicating a relatively high dissolution rate of matrix material which significantly contributes toward drug release. According to this model, there is an inverse relationship between the rate of drug release and decreasing surface area of drug particles; hence it supports our observation about decrease in percentage remaining drug raised to power one-third with time. The model suggests that dissolution controls the release of drugs where structural degradation V-MOF allows for a sustained release profile from DOX encapsulated within it. Thus, kinetic analysis can be interpreted as evidence for surface area reduction being critical toward sustained delivery efficacy regarding DOX@V-MOF supporting zero- and first-order kinetic models results.

The model of Korsmeyer-Peppas kinetic was used to analyze the mechanism of drug release of DOX from the DOX@V-MOF nanocomposite, particularly in cases where several release events may occur at once.^63^ The fractional drug release *vs.* time semi-empirical model is shown in Fig. 6(d). It is used when the exact mechanism of drug release is not known or when different diffusion and relaxation processes are happening at the same time. The release profile shows a continuous sigmoid-shaped curve, which means that there is a slow and controlled release over 120 hours. The release rate constant *K*_F_ was found to be 0.12 h^−1^, and the diffusional exponent *n* was very low at 0.007. This very low *n* value (less than 0.45) designates a Fickian diffusion mechanism, meaning that the drug release is mostly controlled by diffusion through the polymeric or porous matrix with very little help from matrix erosion or swelling. Therefore, doxorubicin (DOX) molecules are mainly released from V-MOF by passing through microchannels or pores inside the CS/PVA nanofiber matrix. The Korsmeyer-Peppas model applies here along with these parameters to further support that this DOX@V-MOF nanocomposite has a diffusion-controlled release profile which agrees with what is seen in zero-order and Hixson-Crowell models while also giving more information on the diffusion mechanisms that affect the release process.

Doxorubicin was released from the DOX@V-MOF nanofiber system through a mechanism that followed diffusion-controlled release. This was analyzed using the Higuchi kinetic model, which is based on Fick's law of diffusion and is most applicable to drug delivery systems based on matrices where drug release happens due to its diffusion through some porous medium. As shown in Fig. 6(e), plotting cumulative percentages of drug release against square root time *t*^1/2^ produced a well-fitting curved profile.^64^ The pattern displays a typical Higuchi release profile, where the amount of drug released is proportionate to the square root of time, suggesting that diffusion is primarily in charge of the process. The Higuchi release constant (*k*_H_) was calculated to be 1.82 which indicates a relatively high diffusion rate of DOX through the CS/PVA matrix incorporated with V-MOFs. The fitting of the Higuchi model to experimental data revealed a good fit underscoring the importance of concentration gradient and porous structure of nanocomposite on DOX release rate. Therefore, using Higuchi model supports that diffusion from DOX@V-MOF is the rate-limiting step in this system, which further proves its potential use for sustained and controlled drug delivery systems where constant diffusion is an essential requirement.

### 3.4. Release behavior of DOX from DOX@V-MOF membrane

**Fig. 7:**
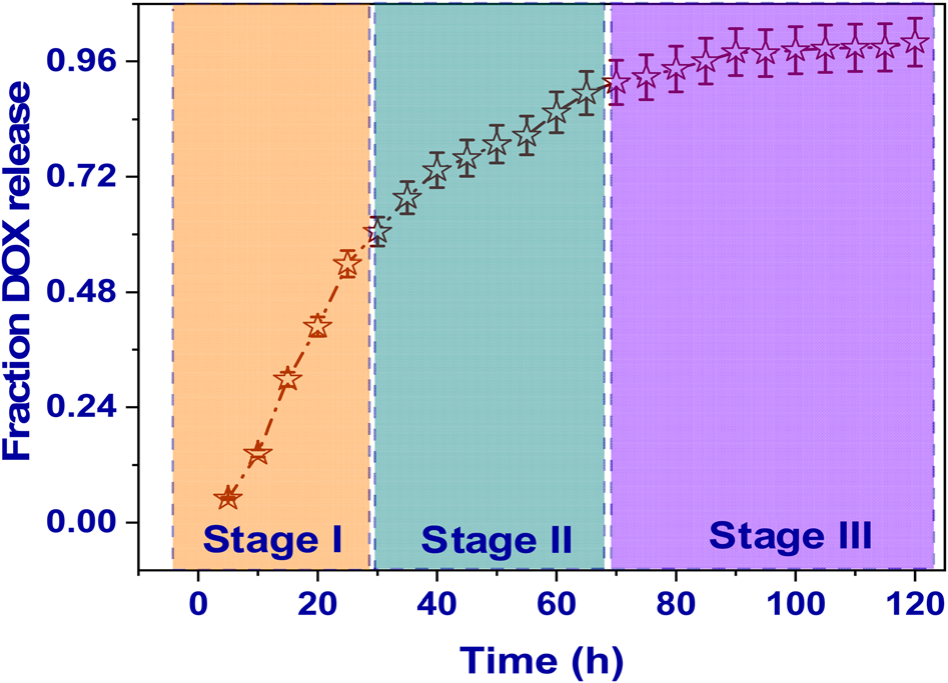
Time-dependent cumulative release profile of DOX from the DOX@V-MOF nanofiber membrane under physiological conditions (pH 7.4, and 37 °C). Error bars characterize standard deviation (*n* = 3).

The DOX@V-MOF membrane showed a total release pattern of DOX that is typically triphasic with three different stages: an initial burst release, then a sustained release phase, and finally a plateau stage.^65^ In the first stage (0–24 hours), very rapid DOX was seen to be released during the first incubation period. This can be attributed to diffusion from loosely bound or surface-adsorbed drug molecules on the outer surface of the nanofiber matrix. In the second stage (24–72 hours), it became more gradual and controlled; this corresponds to sustained diffusion from V-MOF and CS/PVA nanofiber network into external medium. This phase reflects diffusion-driven release from internal pores of carrier matrix into external medium. The last stage had a release profile that was nearing equilibrium and gave off a plateau kind of behavior because there was no more drug available to be released and also because there was no longer a concentration rise among the nanofiber matrix and the medium in which it was supposed to be released (24–72 hours). This triphasic release pattern is proof that the DOX@V-MOF nanofiber membrane can deliver an initial therapeutic dose followed by prolonged and controlled drug release (Fig. 7).

### 3.5. Drug – carrier interaction mechanism

The loading and release performance of DOX in the DOX@V-MOF membrane is based on a grouping of π–π stacking and electrostatic connections between drug molecules and carrier matrix. The aromatic rings in DOX are capable to form π–π interaction with conjugated organic ligands of V-MOF framework which helps in encapsulating the drugs effectively into its porous architecture. Also, electrostatic interaction between amine groups (–NH_3_^+^) protonated DOX and negatively charged functional groups inside the MOF as well as CS/PVA matrix retains the drug under neutral physiological conditions. In acidic media, when these functional groups get protonated, it weakens the electrostatic attraction and consequently increases the diffusion of drugs out from the carrier system. This combined interaction mechanism matches perfectly with what has been observed regarding pH-responsive release behavior for the case of a DOX@V-MOF nanofiber membrane.

### 3.6. Analysis of statistics

#### 3.6.1. ANOVA

With an overall *F*-value of 3036.48 and a *p*-value less than 0.0001, the analysis of variance (ANOVA) data for the DOX release profile from the DOX@V-MOF nanofiber membrane demonstrates the model's significance. This shows that the model has strong statistical significance and predictive ability. Among all independent variables studied, time (B) has been found to be the most important parameter influencing drug release as indicated by its very high *F*-value of 23 712.79.^40^ Temperature (C) and pH (A) are very important, with *F*-values of 1259.49 and 1157.97. Also, the AB interaction term (pH × time), AC (pH × temperature), and BC (time × temperature) were found to be significant at *p* < 0.0001, which means there are very strong synergistic effects among these variables in controlling DOX release. The presence of significant quadratic terms A^2^ and B^2^ indicates a curvature in the response, especially with respect to time; however, C^2^ was not statistically significant. The model has low residual mean square 0.3025 and lack of fit is insignificant (*p* = 0.7085). This indicates that it is a good model that can be relied on. Furthermore, the model has good statistical results with an *R*^2^ value equal to 0.9997, adjusted *R*^2^ equal to 0.9994, and predicted *R*^2^ equal to 0.9959 indicating very high effectiveness in explaining variability in data. Various statistical measures were used to judge the closeness and how well the developed quadratic model can expect results, in addition to the ANOVA results. A low PRESS value indicates a close agreement between observed and predicted responses by the model, thereby confirming the model's ability to predict accurately the behavior of DOX release under different experimental conditions. Similarly, low AIC and BIC values suggest an acceptable trade-off between accuracy and complexity, thus reducing the chances of overfitting. The Adeq Precision value obtained in this study is much greater than the minimum desirable value of 4; hence it indicates adequate model discrimination as well as its applicability in exploring the design space. The proposed response surface model's strength and predicted reliability are validated by all of these statistical metrics. This work shows that pH, temperature, time, and their interactions have a major impact on the release of DOX from the V-MOF nanofiber membrane. Thus, it can be said that this model is appropriate for optimization under drug delivery circumstances (Table S7).

The comparison of the linear, quadratic, cubic, and two-factor interaction (2FI) models fitted to the results from experiments on the release kinetics of DOX from the DOX@V-MOF nanofiber membrane is shown in Table 1. Sums of squares, degrees of freedom (df), mean square, sequential *p*-value, and statistical fit measured by adjusted *R*^2^ and anticipated *R*^2^ were used to evaluate the models. The linear model had a sum of squares equal to 364.51 with mean square set at 40.50, thus providing an initial fit that was quite acceptable having adjusted *R*^2^ value equal to 0.9457 and predicted *R*^2^ equal to 0.9091 but significantly outdone by more complex models introduced later on. The 2FI model enhanced this further by achieving an adjusted *R*^2^ value equal to 0.9924 along with a predicted *R*^2^ value equal to 0.9812 thus proving its ability in accounting interaction effects between independent variables The assessment of every model included critical metrics like sum of squares, degrees of freedom (df), mean square, sequential *p*-value, and statistical fit assessments illustrated through adjusted *R*^2^ and predicted *R*^2^.^66^ The model's validity is confirmed by a very low mean square value of 0.7058 and a highly significant *p*-value (*p* < 0.0001). Due to aliasing issues, the Cubic model will not be further evaluated because there are not enough points to accurately estimate all of the model parameters. Given its good trade-off between complexity and accuracy, our study supports the quadratic model as the most realistic and predictive model for characterizing drug release behavior.

**Table 1:** The number of squares contained in the models given below

Source	Sum of squares	df	Mean square	Sequential *p*-value	Adjusted *R*^2^	Predicted *R*^2^	
Linear	364.51	9	40.50	< 0.0001	0.9457	0.9091	
2FI	39.53	6	6.59	< 0.0001	0.9924	0.9812	
Quadratic	2.12	3	0.7058	< 0.0001	0.9994	0.9959	Suggested
Cubic	0.0000	0			1.0000		Aliased

#### 3.6.2. Cubic interaction and perturbation plot

**Fig. 8:**
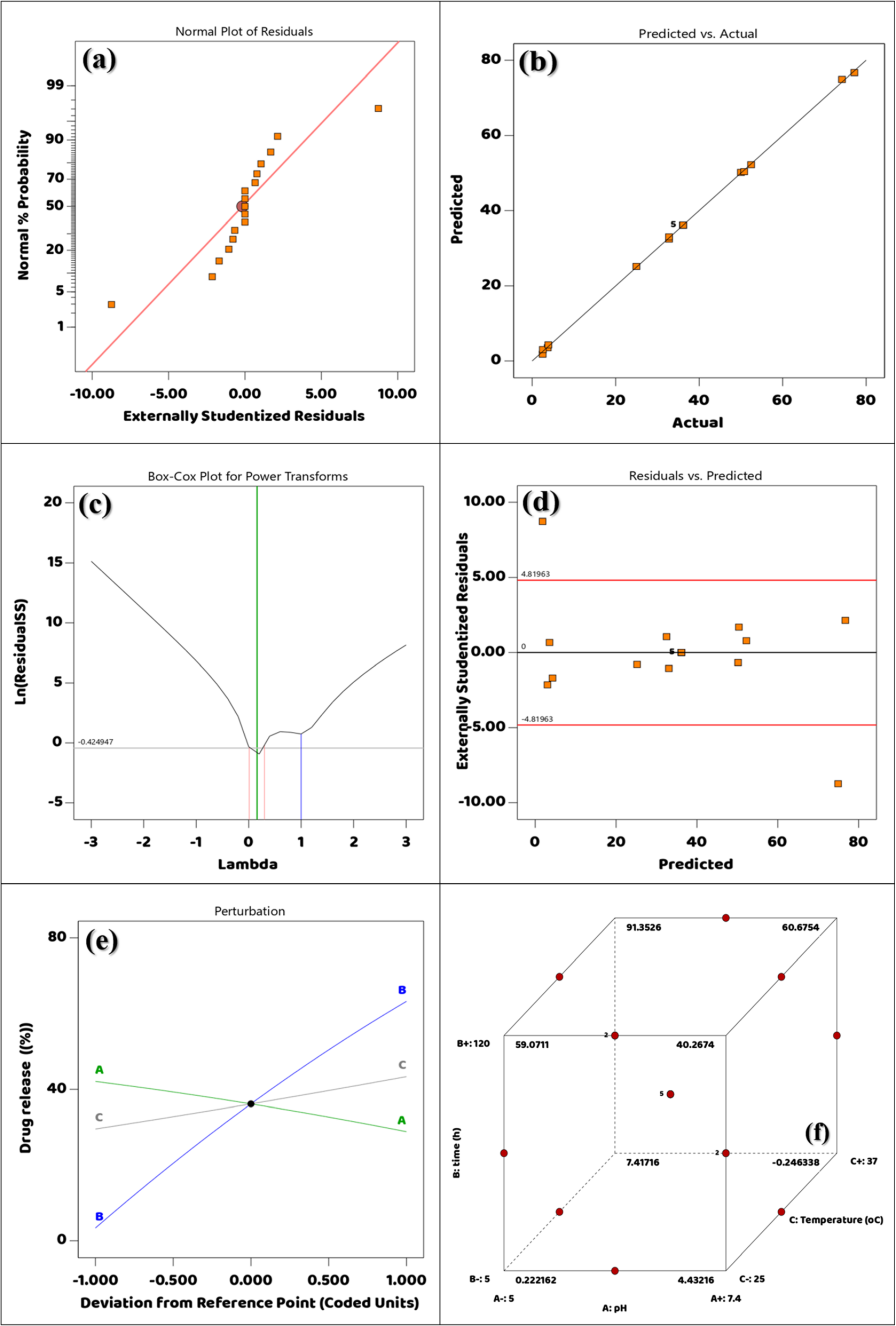
(a) Plotting the correlation between highly standardized residuals with normal % probability, (b) predicted contrasted with actual, (c) Box-Cox Plot for DOX release power transformations, (d) extremely standardized residuals contrasted with predicted, (e) perturbation plot, and (f) Cubic interaction.

The model's suitability in relation to empirical release data will be statistically tested using the plot of normal probability for the residuals associated with the release of DOX from DOX@V-MOF. This graphic shows the externally studentized residuals plotted against the expected normal percentage probability. The majority of orange square data points roughly fit an ideal normal distribution since they come quite near to the red diagonal reference line.^67^ The close fit of the residuals shows a normal distribution, indicating that the release kinetics of DOX from the V-MOF carrier have been sufficiently represented by the kinetic model employed, whether it be zero-order, first-order, Higuchi, or Korsmeyer-Peppas. The small discrepancies observed at the ends of the plot could be due to minor experimental variations or possible outliers; this is not unusual in drug delivery studies that utilize sophisticated nanocarrier systems. Therefore, this statistical analysis supports and strengthens the credibility of the model used to describe controlled and stimuli-responsive release of DOX under physiological conditions as seen in Fig. 8(a).

The Predicted *vs.* Actual graph for DOX release from DOX@V-MOF shows a very good linear fit among the predicted principles from the modeling approach and the experimental data. Here, the *x*-axis represents the experimental DOX release values, and the *y*-axis shows the predicted values from the kinetic model. The orange square data points lie close to the diagonal line, representing a situation where predicted and actual values agree well. This close match indicates high accuracy of this model and suggests it can precisely predict the release kinetics of DOX.^68^ The clear linearity seen indicates that the kinetics of DOX release were well defined, probably in environments sensitive to pH and temperature, thus increasing the model's credibility. These results support the mathematical model's validity for use in controlled and targeted drug delivery since it successfully proves its capability in simulating drug release from the V-MOF-based nanocarrier, as shown in Fig. 8(b).

The figure shows a Box-Cox plot used to discovery the best alteration of the response variable for variance stabilization and improving normality in the data.^69^ This phase is important for improving both the fit and clarity of regression models that describe the release of DOX from DOX@V-MOF. The graph displays the natural logarithm of the leftover sum of squares (Ln (Residual SS)) on the *y*-axis and various values for the transformation variable lambda (*λ*) on the *x*-axis. The curve shown reflects changes in the residual sum of squares as a function of different *λ* values. The lowest point on this curve gives us the best *λ*, which tells us when variance is most efficiently stabilized and residual error reduced. In this case, an ideal value for *λ* has been found to be about −0.42 based on what is marked on the curve; therefore, inverse square root or logarithmic function transformations could help make normality more pronounced in our dataset. The vertical lines in the plot denote a 95% confidence interval around the optimal *λ*; since zero falls within this interval, a logarithmic transformation would be appropriate statistically. This analysis implies that original DOX release data might benefit from such a transformation to better meet assumptions related to linear modeling and eventually help achieve more profound insight into and prediction capabilities regarding drug release dynamics from V-MOF-based nanofiber systems as shown in Fig. 8(c). The diagnostic plots in Fig. 8(a–c) were used to check if the proposed regression model was valid and adequate. The residuals are normally distributed and there is no significant systematic variation in the model predictions, according to the normal probability plot of the residuals, which revealed that the data points were almost on a straight line. The accuracy of the proposed model was confirmed by the very strong agreement between the experimental and model-projected values of DOX release in the predicted *vs.* real plot. In addition, from the Box-Cox plot it can be inferred that no substantial data transformation is necessary; hence it validates that original response data is appropriate for regression analysis.

Residuals *vs.* Predicted the plot in this figure is used as a diagnostic for checking the adequacy of the regression model used to describe the release behavior of DOX from DOX@V-MOF and whether its assumptions are satisfied. This is a scatter plot through predicted values of DOX release by the fitted model on the *x*-axis and externally studentized residuals on the *y*-axis; these residuals indicate how much each individual data point affects the whole model. In order to demonstrate homoscedasticity and the absence of systematic error in prediction, residuals should be dispersed randomly about the zero line.^70^ The common of the orange square data points are clustered around the zero-horizontal centerline with just a few scattered beyond the red control limits (±4.81963), indicating few potentially influential outliers. The absence of any discernible pattern among the data and the overall horizontal distribution of residuals provide additional support for assumptions regarding model linearity and random error distribution. Consequently, this graphical representation serves to evaluate how well the model describes DOX release while acknowledging that some outlier values may be due to experimental variation or biological factors affecting drug release mechanisms (Fig. 8(d)).

The perturbation plot represents the relative impacts of three coded variables, namely A (pH), B (Time), and C (Temperature), on the percentage of drug release from the system of DOX@V-MOF. The changes from the situation formulation in implied units are indicated on the *x*-axis while the corresponding percentages for drug release are indicated on the *y*-axis. From this plot, it is very much evident that variable B has the highest effect on DOX release; it has a steep positive slope which means increasing B will lead to a large increase in drug release thus proving its significance as an important factor for enhancing performance in this formulation.^71^ Variable A has a small negative slope indicating that increasing pH is associated with a slight decrease in drug release whereas variable C has a near-zero slope indicating no significant effect on the release profile over this range. The central black dot represents the reference formulation which is used here to compare the effects of each variable. This analysis underscores the critical importance of optimizing variable B in the process of designing and developing an actual stimuli-responsive DOX delivery system based on DOX@V-MOF nanofiber membrane, as exemplified in Fig. 8(e).

Three-dimensional cube plot showing how the three most significant variables—pH (A), time (B), and temperature (C)—interact to affect the percentage of DOX released from the DOX@V-MOF nanofiber system. The cube plot shows the low (−) and high (+) values for each factor. The experimental data points are represented by red spheres, with the corresponding release percentages annotated within the plot.^71^ Notably, the highest level of drug release (91.35%) occurs at conditions of low pH (5.0), a long release time (120 hours), and high temperature (37 °C), which simulate the microenvironment within tumor tissues. The lowest release rate is approximately −0.25% at neutral pH (7.4), short release time (5 hours), and low temperature (25 °C), which represent physiological conditions meant to inhibit the early diffusion of DOX. All intermediate values are allocated within the experimental design space, hence proving how significantly each variable influences the drug release dynamics. This analysis proves that this system is sensitive to changes in pH, time, and temperature; thus, it confirms its design as a stimuli-responsive DOX delivery system for controlling and targeting therapeutic releases in cancer treatment as shown in Fig. 8(f). The perturbation and three-dimensional response surface plots in Fig. 8(e and f) demonstrate the individual and combined effects of pH, temperature, and contact duration on the percentage release of DOX from the DOX@V-MOF nanofiber membrane. The curvature that was observed in the response surfaces confirms that significant interaction effects exist between the studied variables, which is also consistent with the statistical significance of the interaction terms obtained from ANOVA analysis. These plots provide a visual representation of an optimized experimental domain as well as demonstrating combined influences by process variables on performance for drug release.

#### 3.6.3. Checking for model adequacy

**Fig. 9:**
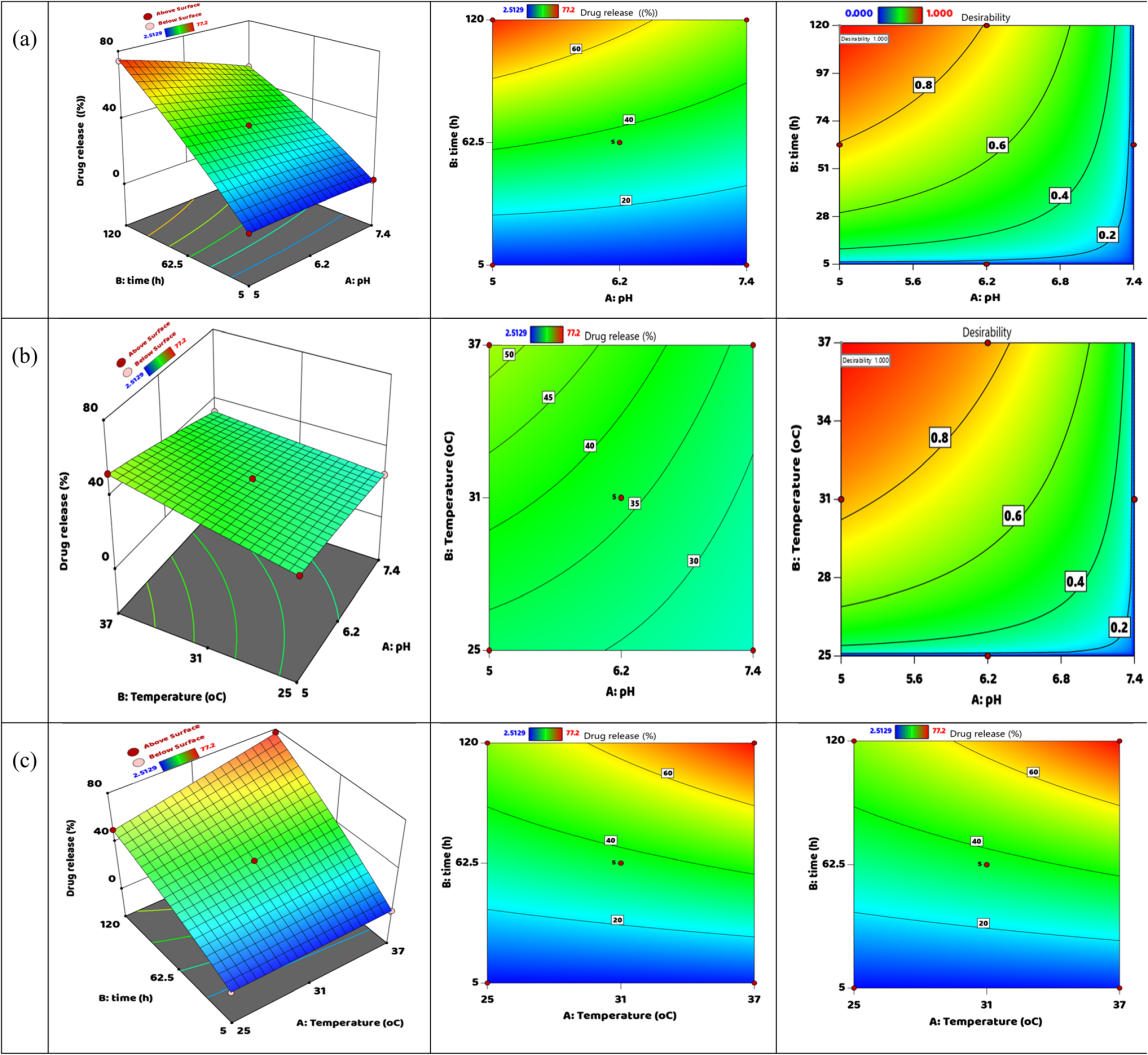
The release of DOX from DOX@V-MOF nanofiber membrane: contour, three-dimensional interaction, and desirability (a) pH and time, (b) pH and temperature, (c) temperature and time.

A big group of the orange square data points is found near the zero-horizontal centerline in this study. The three-dimensional surface plot seen on the left reveals that drug release grows with longer times and under acidic conditions. The release reaches high levels, peaking at about 77.2% at a pH of 5 after 120 hours, which simulates conditions in the tumor microenvironment.^72^ This observation noted the system's increased sensitivity to variations in pH and its temporal dependence concerning drug delivery. The intermediate contour plot demonstrates this relationship in a two-dimensional framework where smooth gradient zones are used to describe how lower pH values and longer durations act synergistically toward enhanced drug release. This overhead visualization makes it easy to see what the ideal formulation parameters would be. The desirability plot on the right, however, takes both factors into an optimization framework by showing how different combinations influence the overall efficiency of the system in reaching maximum drug release. The red area, with a desirability score of 1.0, proves that optimal release conditions are low pH and long times; hence, all these graphical representations support that DOX@V-MOF system has strong sensitivity to acidic pH condition and long release time which means it is good for targeted as well as controlled delivery of cancer therapeutics revealed in Fig. 9(a).

The three graphs in the figure show how temperature (B) and pH (A) work together to affect how much DOX comes out of the DOX@V-MOF membrane system. The 3D surface scheme on the left shows a flat surface, meaning that, for the conditions tested (pH between 5 and 7.4 and temperatures from 25 to 37 °C), changes in temperature have little effect on DOX release compared to changes in pH.^73^ The minor variations in drug release were 30 to 40%, indicating a system that is responsive to pH changes. Temperature, on the other hand, appears to have little influence under the specific experimental conditions. This is consistent with the central contour plot, which predominantly shows green areas with only slight changes in release amounts, underscoring the negligible effect of temperature on drug diffusion. On the other hand, the desirability plot on the right gives good information about the optimization process showing that at acidic pH and higher temperature of 37 °C, maximum desirability shown by a value of 1.0 is reached. However, it should be pointed out that the rate of release does not really speed up over time.^74^ The findings suggest that temperature might slightly influence the acceleration of drug release, perhaps through softening or swelling the nanofiber matrix. However, the key factor controlling the DOX release from the V-MOF platform is the acidic pH environment. Therefore, this system shows main sensitivity to pH changes and secondary sensitivity to temperature changes for better drug delivery efficiency (Fig. 9(b)).

The pictures show how temperature (A) and time (B) work together to change the speed at which DOX comes out of the DOX@V-MOF membrane system.^74^ The three-dimensional surface plot (left) discloses a clear positive interaction among drug release and both temperatures increase and extended-release time, with a maximum value of about 77.2% at higher temperatures (37 °C) over a release period of 120 hours. This finding suggests that, although pH is the main variable, temperature has an important influence on DOX diffusion from the carrier matrix, possibly due to increased mobility of polymer chains or partial degradation of the V-MOF. The corresponding contour plot (middle) further supports this interaction by showing that there is an ongoing increase in drug release along both dimensions of temperature and time. The smooth contour lines in this plot demonstrate the extent of interaction between these two factors on the release of DOX. The desirability plot (right) depicts the optimization zone, which indicates that most favorable conditions exist when desirability approaches 1.0 at high temperatures and long release intervals. All these graphs clearly illustrate that although pH is still the predominant controlling factor, it is temperature and time working in conjunction that enable a controlled and efficient drug release profile within the DOX@V-MOF smart delivery system as shown in Fig. 9(c).

Fig. 9 presents the three-dimensional response surface, contour, and desirability designs that visualize how pH, temperature, and contact time interact to affect the release efficiency of DOX from the DOX@V-MOF membrane. As seen in the response surface plot presented in Fig. 9(a), a significant synergistic interaction between pH and contact time is evident because increasing the incubation time under acidic conditions leads to a marked increase in the percentage of drug released; this indicates that diffusion of DOX is enhanced at lower pH values. In Fig. 9(b), for the interaction between pH and temperature, it is observed that release proceeds more readily at higher temperatures particularly in acidic media due to increased molecular mobility as well as weakened interactions between drug and carrier matrix. Likewise, Fig. 9(c) demonstrates that an increasing trend in drug release efficiency occurs with increasing values of both temperature and contact time which further confirms the diffusion-controlled nature of the release mechanism. These contour plots correspondingly assert significant interaction effects among all studied variables while desirability plots designate optimal DOX release at low pH, high temperature, and long contact time; thus, underscoring consistent ANOVA results regarding environmental condition importance in controlling DOX@V-MOF nanofiber membrane release performance.

#### 3.6.4. Model validation and the desirability approach

**Fig. 10:**
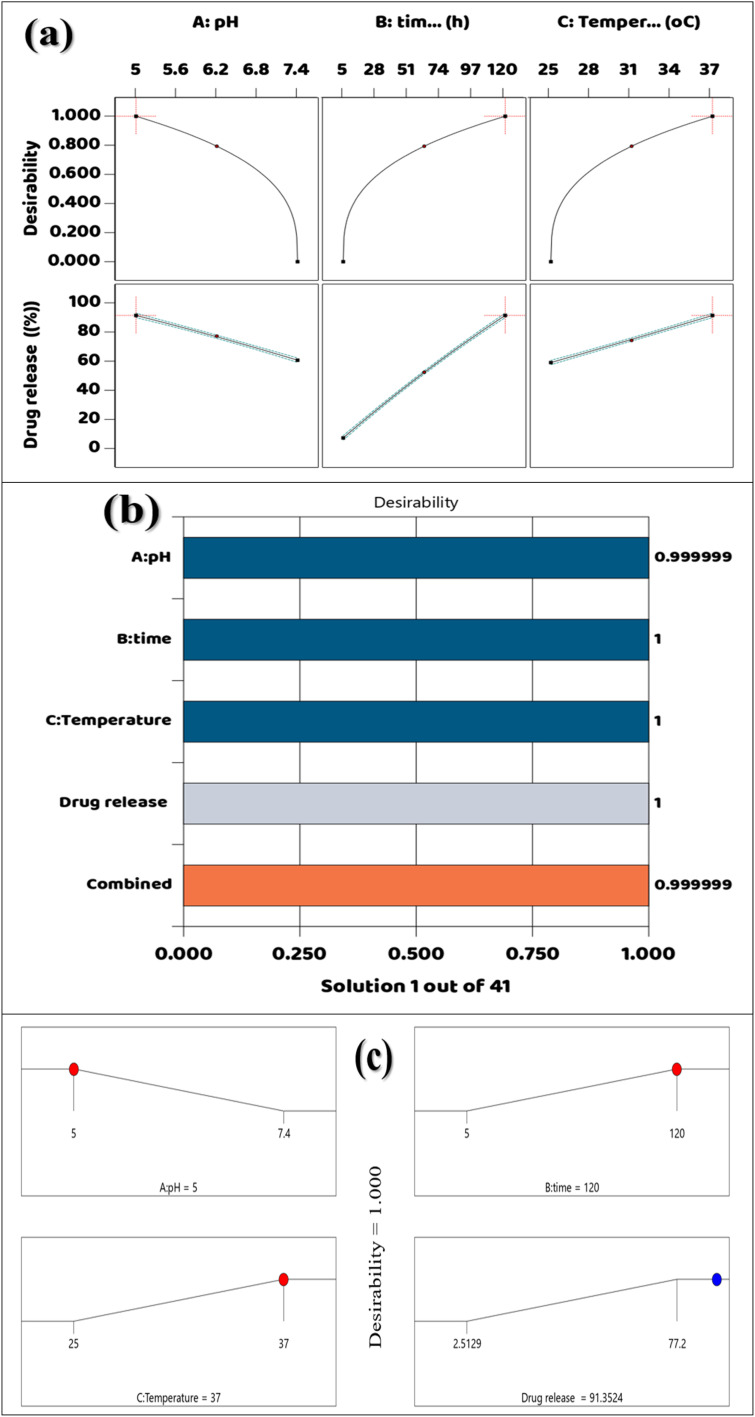
(a) The growing interest in the optimal numerical solutions, (b) individual desirability is displayed as a bar graph, and (c) desirability of every solution.

The individual effects of pH (A), time (B), and temperature (C) on the release percentage of DOX from the DOX@V-MOF nanofiber matrix as well as its inherent attraction are displayed in the optimization and prediction profiler graphs shown in Fig. 10(a).^72^ Desirability curves that show how each parameter affects the optimization target are included in the study's upper section. A maximum desirability (value = 1.0) is achieved at minimum pH (5.0), maximum release time (120 h), and high temperature (37 °C), indicating that these combined conditions yield an optimal scenario for enhanced drug release performance. The desirability decreases sharply with increasing pH and increases strongly with longer release times, clearly demonstrating the significant roles of pH sensitivity and exposure time. The temperature curve shows a steady increase, further emphasizing its crucial role in promoting drug release. Expected percentages of drug release associated with these variables are presented in the lower section; these confirm established trends: DOX release increases with longer time and higher temperature but decreases with increasing pH. The effect of these trends is supported by prediction bands enveloping each curve. Such predictions reveal that acidic pH, long times, and moderate hyperthermia can maximize DOX release and desirability, thereby enhancing responsiveness and intelligence in the DOX@V-MOF system.

The desirability bar chart is a great view of the best way to increase the DOX release from DOX@V-MOF membrane drug delivery system, based on a multi-variable optimization model. This graph displays the individual and total desirability scores for each of the following variables: pH (A), time (B), and temperature (C), as well as the treatment's overall efficacy and predicted medication release.^75^ Both temperature and drug release have achieved a maximum desirability score of 1.000000, representing that these two variables are completely optimized under the conditions set. The parameters of pH and time show very high desirability notches of 0.999999 and 0.999998, individually, representing their almost optimal performance. An overall combined desirability notch of 0.999999 further approves that this solution, which is ranked first out of 41 solutions evaluated, is very good at achieving the desired profile for drug release. This analysis thus concludes that under optimal conditions characterized by low pH, long release time, and increased temperature, the system DOX@V-MOF runs at its best possible performance for applications in controlled and targeted anticancer drug delivery as shown in Fig. 10(b). A comprehensive overview of the optimization objectives, key variables, and desirability assessments for the DOX release from DOX@V-MOF nanofiber membrane is presented (Table 2).

**Table 2:** A comprehensive overview of the optimization objectives

Limit/response	Optimization goal	Importance weight	Desirability notch
A: pH	Within range (5–7.4)	1	0.999999
B: time (h)	Within range (5–120)	1	0.999998
C: temperature (°C)	Within range (25–37)	1	1
DOX release (%)	Maximize	5	1
Combined	—	—	0.999999

Fig. 10(c) shows an optimization profile for the DOX@V-MOF drug delivery system. It summarizes the conditions that need to be met to get the maximum release of doxorubicin (DOX). Each subplot talks about a different variable: pH (A), time (B), temperature (C), and drug release. It explains how each factor affects the total results. The red and blue markers show the chosen optimal values for each variable, corresponding to a peak global desirability score of 1.000. Results indicate that a pH of 5 is optimal for formulation parameters, as well as 120 hours of release time at 37 °C - conditions which replicate those found in tumor tissue since they are both acidic and warm. Under this condition, the predicted release of the drug would be 91.3524% as indicated by the blue marker. This further proves that this system is highly sensitive to variations in pH, time, and temperature thereby confirming its ability to control and effectively deliver anticancer drugs when required under similar circumstances. The optimization profile shows clearly that the conditions described create a situation that is best for increasing therapeutic effect and minimizing any drug waste or accidental releases.^75^

### 3.7. The biological process

#### 3.7.1. Anti-cancer characteristics

**Fig. 11:**
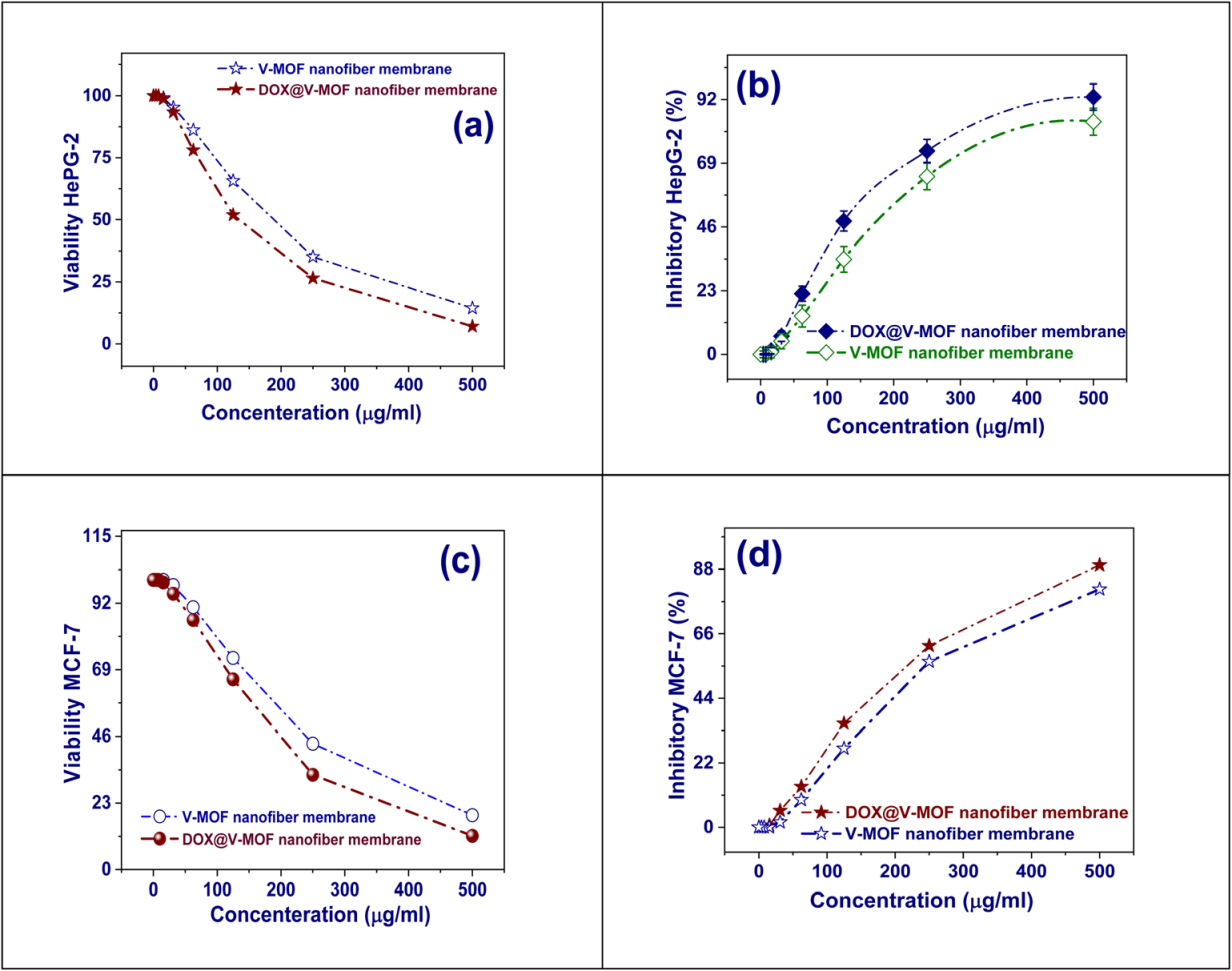
V-MOF membrane effectiveness, DOX@V-MOF membrane, (a) HePG-2 cell viability, (b) HePG-2 inhibitory (%), (c) MCF-7 cell viability, (d) inhibitory of MCF-7 (%).

The cytotoxicity test on V-MOF membranes and DOX@V-MOF membranes was conducted using HepG-2 liver cancer cells. Cell viability and inhibition percentage analyses were performed with a clear dose-dependent relationship. The viability graph in Fig. 11(a) indicates that both membrane types consistently reduced the viability of HepG-2 cells with increasing concentration.^76^ The cytotoxicity results of the DOX@V-MOF nanofiber membrane was significantly higher than those of all concentrations tested. At the highest dose of 500 µg mL^−1^, cell viability dropped below 20% with the use of a DOX@V-MOF membrane. Relatively low cytotoxicity was displayed by the V-MOF membrane; hence, increased anticancer activity can be attributed to doxorubicin's presence. This is further supported by inhibition percentage data in Fig. 11(b), where the DOX@V-MOF membrane had an inhibition rate nearing 90% at a concentration of 500 µg mL^−1^; however, there was less inhibition with the V-MOF membrane but still significant levels. These results together strongly support that loading doxorubicin into the V-MOF matrix increases the anticancer activity of the nanofiber membrane particularly in terms of its ability to inhibit HepG-2 cell proliferation in a concentration-dependent manner.^77^

The cytotoxicity evaluation of V-MOF membrane and DOX@V-MOF membrane on MCF-7 breast cancer cells is carried out *via* cell viability assessments, as illustrated in Fig. 11(c) with corresponding inhibition percentages presented in Fig. 11(d). The results reveal that both nanofiber formulations reduce the survival rate of MCF-7 cells in a concentration-dependent manner. More specifically, at lower concentrations (*e.g.*, 50–100 µg mL^−1^), cell viability for both membrane types remains relatively high.^78^ An increase in concentration is associated with a steep drop in cellular survival rates. The DOX@V-MOF membrane shows a more significant reduction in cell viability compared to the V-MOF membrane alone, which means that it has increased cytotoxicity due to the presence of doxorubicin, a chemotherapeutic agent. At the maximum concentration verified (500 µg mL^−1^), cell viability for the formulation loaded with DOX falls to about 10%, while the V-MOF sample shows slightly higher viabilities between 20% and 25%. The inhibition percentages graph confirms these results by showing more inhibitory effects for both formulations as their concentrations increase.^79^ The DOX@V-MOF membrane shows better inhibition properties, achieving almost 90% inhibition at 500 µg mL^−1^ compared to the 75–80% inhibition observed with the V-MOF nanofiber. This proves that the DOX loading into the structure of the MOF membrane increases anticancer activity by significantly raising cytotoxicity and showing a dose-dependent inhibition of MCF-7 cell growth.

#### 3.7.2. Antioxidant activities

**Fig. 12:**
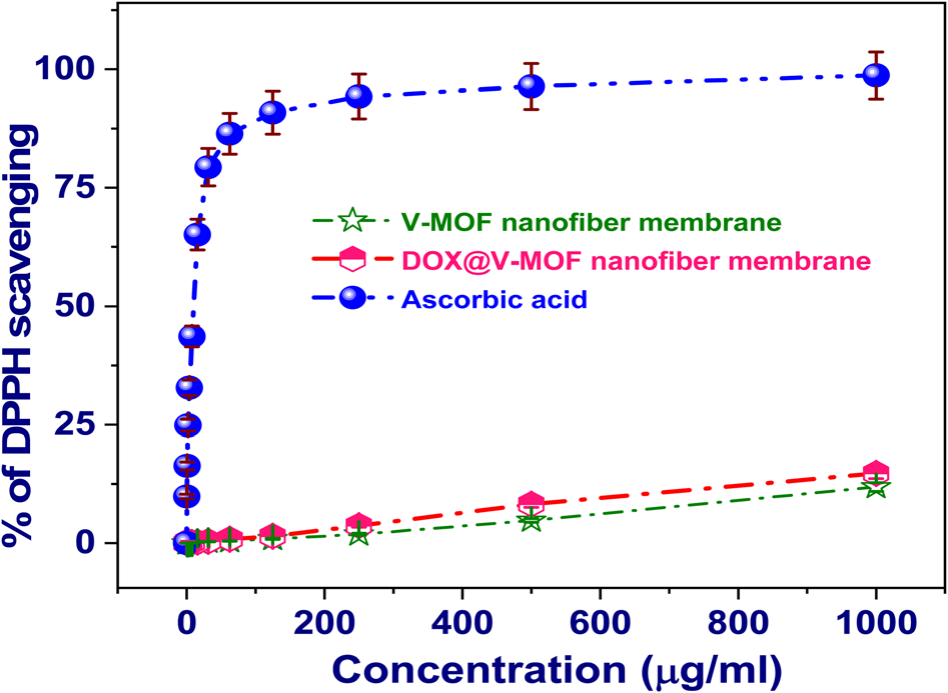
V-MOF membrane and DOX@V-MOF membrane scavenging of DPPH radicals.

The capacity of V-MOF membrane and DOX@V-MOF membrane to quench DPPH radicals was tested using ascorbic acid as the standard antioxidant. The results showed that ascorbic acid had a strong and fast antioxidant effect with radical scavenging capacity over 90% at less than 100 µg mL^−1^, proving its high efficiency in scavenging free radicals. On the other hand, V-MOF and DOX@V-MOF membranes' antioxidant activities were much lower; there was an increasing trend in DPPH scavenging with rising concentrations.^80^ The V-MOF nanofiber membrane exhibited less than 15% DPPH radical scavenging activity at the highest dose tested, 1000 µg mL^−1^. The performance of the DOX@V-MOF membrane indicated a slight improvement with scavenging activity of about 20%. This improvement is due to doxorubicin being added since it has functional groups like quinones and hydroxyls that can donate electrons or hydrogen and increase the ability to scavenge radicals.^81^ Nanofiber membranes exhibit lower antioxidant capacities than ascorbic acid. However, a slight increase in the DOX-loaded membrane indicates a potential additional benefit for applications in oxidative stress management, especially regarding cancer treatment or wound healing therapies (Fig. 12).

#### 3.7.3. Antibacterial activities

**Fig. 13:**
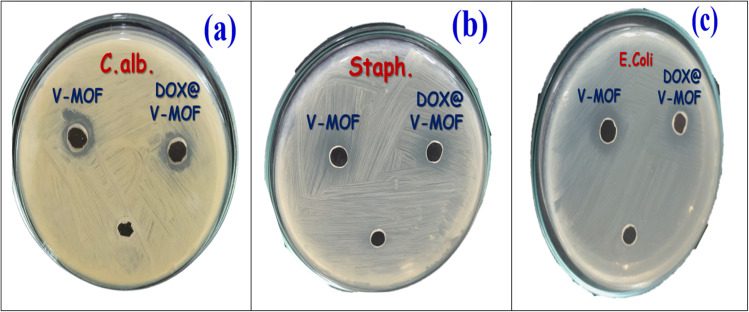
Inhibition zones for V-MOF membrane, DOX@V-MOF membrane to: (a) *C. albicans,* (b) *S*. *aureus,* and (b) *E. Coli*.

By assessing inhibitory zone widths, the antibacterial and antifungal properties of V-MOF and DOX@V-MOF were evaluated against the fungal strain *Candida albicans*, the Gram-positive bacterium *Staphylococcus aureus*, and the Gram-negative bacterium *Escherichia coli* (Table 3). In isolation, V-MOF showed moderate antimicrobial activity with inhibition zones of 10 mm for *S. aureus*, 12 mm for *E. coli*, and 11 mm for *C. albicans* as seen in Fig. 13(a). When DOX was loaded into the V-MOF matrix to form DOX@V-MOF, it exhibited much better antibacterial performance with increased inhibition zone diameters of 13 and 14 mm against *S. aureus* and *E. coli*, individually.^82^ This finding indicates that a synergistic interaction between the chemotherapeutic agent and the MOF has been achieved successfully. There was no enhancement, however, in antifungal efficacy against *C. albicans*; inhibition zones were both equal to 11 mm for V-MOF and DOX@V-MOF, which meant that DOX has almost no effect on antifungal activity (Fig. 13(b)). The standard antibiotic gentamicin showed much better activity with inhibition zones of 24, 30, and 20 mm against *S. aureus*, *E. coli*, and *C. albicans*, individually. From these results, it can be seen that even though DOX@V-MOF shows better antibacterial property compared to unmodified MOF, its antifungal property does not change and still has a lower overall effect compared to normal antibiotics (Fig. 13(c)).

**Table 3:** The antimicrobial efficacy of DOX@V-MOF and V-MOF membranes was tested against *Staphylococcus aureus*, *Escherichia coli*, and *Candida albicans*

Sample	Gram positive bacteria	Gram negatvie bacteria	Fungi
	*Staphylococcus aureus* (RCMB 010010) ATCC 25923	*Escherichia coli* (RCMB 010052) ATCC 25955	*Candida albicans* (RCMB 005003 (1) ATCC 10231
V-MOF	10	12	11
DOX@V-MOF	13	14	11
Regulator (*Gentamycin*)	24	30	20

### 3.8. Study limitations and the process of drug loading

Although the study's results are very promising, a number of limitations highlight the need for more investigation into the underlying molecular pathways. It should be mentioned that all evaluations were conducted *in vitro*, which is insufficient to replicate the intricate physiological environment found *in vivo*. These trials do not take into account variables like immunological reactions, enzymatic breakdown, and variations in blood circulation.^83^ Therefore, further research with animal models is necessary to verify the effectiveness and biocompatibility of the medicines in question.^84^ Furthermore, the nanofiber membrane's hemocompatibility, long-term stability, and potential immunogenicity have not yet been assessed, which poses significant obstacles to its usage in clinical settings. Additionally, the approach lacks active targeting techniques that could improve selectivity toward tumor tissues, such as ligand-functionalized surfaces. Additionally, because of the electrostatic repulsion between the positively charged DOX and the V-MOF surface, the procedure of loading mechanism of DOX depends on post-synthesis adsorption, which may restrict the process's effectiveness and dependability.^85^ According to our understanding of the mechanism, a number of non-covalent interactions seem to confine DOX molecules inside the V-MOF's structure. These interactions may diminish in acidic environments (pH 5.0), allowing the medication to be released. However, the binding strength increases at neutral pH, which promotes a longer release time.^86^ By interacting with bacterial membranes, proteins, and DNA, vanadium ions (V^3+^) will be doped in the MOF to provide structural stability and antibacterial action. Diffusion across the hydrated polymer network and a release mechanism dependent on matrix degradation over time are both supported by the CS/PVA nanofiber matrix. Because V^3+^ is redox-active and tumor cells have an internal reducing environment, DOX may be released by MOF degradation, increasing the system's responsiveness to stimuli.^87^ The techniques and limitations demonstrate how urgently this multipurpose nanoplatform has to be enhanced and thoroughly assessed physiologically (Tables S8 and S9).

### 3.9. Limitations and future perspective

Though the DOX@V-MOF nanofiber membrane showed good results in drug release tests and biological activity *in vitro*, some limitations must be pointed out. Vanadium-MOFs are known to be redox-active and may have antioxidant properties, but this study looked mainly at them as a structural carrier for controlled drug delivery; it did not use any specific therapeutic mechanisms that involve Vanadium redox mediation. The biological tests included only *in vitro* cytotoxicity and antimicrobial activities, with no evaluation *in vivo* or active targeting strategy applied here. Also, before clinical use, vanadium-containing nanomaterials' potential toxicity and long-term biosafety should be assessed. Future work will thus emphasize *in vivo* validation, targeted functionalization of the nanofiber membrane, and mechanistic exploration of V-mediated redox interactions within tumor microenvironments to enhance therapeutic efficacy and safety.

## 4. Conclusion

Herein, a novel smart drug delivery system based on doxorubicin (DOX) encapsulated into vanadium metal–organic framework (V-MOF) and embedded within chitosan/polyvinyl alcohol (CS/PVA) electrospun nanofiber membrane was developed. It showed high drug loading capacity and encapsulation efficiency as well as excellent physicochemical stability confirmed by FTIR, XRD, SEM, EDX, XPS, and BET surface area characterization. *In vitro* studies of drug release revealed pH- and temperature-responsive dual release kinetics with a significant increase in DOX release at pH 5.0 and 37 °C that mimics the tumor microenvironment. Kinetic modeling of drug release using zero-order Higuchi and Korsmeyer-Peppas models suggested a predominantly diffusion-controlled and swelling-regulated mechanism. Biological assessments validated the multifunctionality of the system through potent anticancer activity against MCF-7 and HepG-2 cell lines along with antioxidant and antimicrobial activities for the DOX@V-MOF nanofiber system. Further optimization *via* Box-Behnken statistical modeling provided optimal formulation conditions to maximize drug release as well as system efficacy. The research highlighted the possible use of the DOX@V-MOF membrane as a flexible and effective intelligent system for delivering drugs directly to cancer sites. It focused on treatment with controlled release, reduced side effects, and added therapeutic advantages that could be used in future specific applications of nanomedicine in clinical settings.

## Author contributions

Ahlem Guesmi: conceptualization, data curation, investigation, validation, visualization, writing – review and editing. Naoufel Ben Hamadi: conceptualization, data curation, investigation, resources, validation, visualization, writing – review and editing. Wesam Abd El-Fattah: conceptualization, data curation, investigation, visualization, writing – review and editing. Mohamed G. El-Desouky: conceptualization, data curation, investigation, methodology, validation, visualization, writing – original draft. Ashraf A. El-Bindary: conceptualization, data curation, investigation, resources, validation, visualization, writing – original draft, writing – review and editing.

## Conflicts of interest

There are no conflicts of interest to declare.

## Supplementary Material

RA-016-D6RA01018K-s001

## Data Availability

The data that support the findings of this study are available from the corresponding author upon reasonable. Supplementary information (SI) is available. See DOI: https://doi.org/10.1039/d6ra01018k.
