# Supplementary material for: Electrospun nanofiber membrane of chitosan/polyvinyl alcohol embedded with DOX-loaded V-MOFs for controlled drug release and multifunctional biological activity
Source: RSC Adv. 2026 Apr 1;16(20):17650–75. doi: 10.1039/d6ra01018k (PMC13040670; doi:10.1039/d6ra01018k)
Supplement: RA-016-D6RA01018K-s001 [file RA-016-D6RA01018K-s001.pdf]

Supplementary Information (SI) for RSC Advances.
This journal is © The Royal Society of Chemistry 2026

Electrospun nanofiber membrane of chitosan/polyvinyl alcohol embedded with DOX-loaded V-MOFs for controlled drug release and multifunctional biological activity

Table S1. Chemical name, formula, and company.

Chemical name	Formula	Company
Chitosan	$C_{56}H_{103}N_9O_3$	Sigma-Aldrich, Germany
Polyvinylalchol	$(C_2H_3O)_n$	Sigma-Aldrich, Germany
Vanadium chloride	VCl_3	Sigma-Aldrich, Germany
benzene-1,3,5-tricarboxylic acid	$C_6H_3(CO_2H)_3$	Sigma-Aldrich, Germany
Methanol	CH_3OH	LOBA CHEMIE PVT.LTD, India
Ethanol	C_2H_6O	Sigma-Aldrich, Germany
Sodium hydroxide (99%, AR)	NaOH	Chimmed, Russia
Hydrochloric acid (37%, AR)	HCl	LOBA CHEMIE PVT.LTD, India

Table S2. Instruments and equibments.

Test name	Abbrevation	Instrument name	Company	Illustration
Fourier transformer infrared	FT-IR	A Nicolet IS10 Fourier transform infrared (FTIR) spectrometer	Thermo Fisher Scientific, Waltham, MA, USA	equipped with an attenuated total reflectance accessory and which ran in the 4000-400 cm^{-1} range was used to gather FTIR spectra
Powered X-ray diffraction	PXRD	Siemens diffractometer (model D500, Germany)	Germany	patterns were captured from powder samples through the use of a Siemens diffractometer (model D500, Germany) that was fitted with a Cu-K radiation source (wavelength 1.54 Angstroms (Å)) operating at 30 kV and 20 mA.
Scanning Electron Microscope	SEM	(JSM-6510LV, JEOL Ltd., Tokyo, Japan)	JEOL Ltd., Tokyo, Japan	The morphology of the investigated sorbents was analyzed with the use of a scanning electron microscope
X-ray photoelectron spectroscopy	XPS	K-ALPHA (Themo Fisher Scientific, USA)	Themo Fisher Scientific, USA	Used for determination the elemental analysis for the compound
Braunnar Emmet Teller	BET	Quantachrome Instruments, Anton Paar Quanta Tec, Inc., Boynton Beach, FL,	Quanta Tec, Inc., Boynton Beach, FL, USA	was utilised for surface and pore analysis (Brunauer Emmett-Teller (BET) surface area, porous volume, and pore size), and NovaWin Software (v11.0) was used for data interpretation.

		USA		The BET surface area of material adsorbents was obtained by the application of nitrogen adsorption-desorption isotherms at 77K through the use of a specific analyser (Quadrasorb–EVO, Quantachrome, USA).
UV-visible spectrophotometer	UV spectrophotometer	Perkin-Elmer AA800 spectrophotometer Double beam, with 1 cm cell length.		Measuring the concentration of the adsorbate soulution via using bear lambert law
Energy Dispersive X-ray	EDX	Leo1430VP microscope	Carl Zeiss AG, Jena, Germany	Elemntal analysis of the material
Transmission electron microscopy	TEM	TEM, FEI Teanci G2 F20, USA	FEI Teanci G2 F20, USA	Determination the morphology of the material and size
pH meter	pH	HANNA (model 211)	USA	Measuring the acidity or basicity of the solution
Sonication	Ultrasonic	Elmasonic P300H ultrasonic bath, continuous mode, power 380 W	Elma Schmidbauer GmbH, Singen, Germany	Sonication of the material as well as used ton disperse material on the solution as it decrease the particle size of the material
Water bath	Shaking	GFL Orbital Shaker 3017		

Table S3. True variables, codes, and their BBD levels.

Code	Variables	-1	0	+1
A	pH	5	6.2	7.4
B	Temperature (^oC)	25	33.5	42
C	Time (h.)	5	52.5	100

Table S4. Investigate the percentage of DOX release and the surface interactions.

Run	Actual variables			DOX release (%)		
	pH	Time (h)	Temperature (^oC)	Investigational	Predicted	Residue
1	6.2	62.5	31	36.179	36.18	0.0000
2	6.2	62.5	31	36.179	36.18	0.0000
3	6.2	5	25	2.556	3.03	-0.4786
4	6.2	62.5	31	36.179	36.18	0.0000
5	5	62.5	37	52.46	52.24	0.2219
6	6.2	5	37	3.88	4.29	-0.4128
7	5	62.5	25	32.7875	32.50	0.2877
8	5	5	31	3.73	3.54	0.1909
9	6.2	120	37	77.2	76.72	0.4786
10	6.2	120	25	50.7895	50.38	0.4128
11	6.2	62.5	31	36.179	36.18	0.0000
12	7.4	5	31	2.5129	1.81	0.7005
13	7.4	62.5	37	32.78	33.07	-0.2877
14	6.2	62.5	31	36.179	36.18	0.0000
15	7.4	62.5	25	24.981	25.20	-0.2219
16	7.4	120	31	50	50.19	-0.1909
17	5	120	31	74.2308	74.93	-0.7005

Table S5. Equations used in this work to fit the data of adsorption experiments.

Serial	Equation	Nmae	Description	Ref.
1	$Q_o^{1/3} - Q_t^{1/3} = K_{HC}.t$	Hixson–Crowell model	Q_0 = Initial amount of drug<br>Q_t = Remaining amount of drug at time t<br>K_{HC} = Hixson–Crowell dissolution rate constant<br>t = Time	[1]
2	$Q_t = Q_0 + K_0.t$	Zero-Order	Q_t = Amount of drug released at time ttt<br>Q_0 = Initial amount of drug in the solution (often 0)<br>K_0 = Zero-order release constant (units: concentration/time)<br>t = Time	[2]
3	$\ln Q_t = \ln Q_0 - K_1 \cdot t$	First order	Q_0 = Initial amount of drug<br>Q_t = Amount of drug remaining at time ttt<br>K_1 = First-order rate constant (1/time)<br>t = Time	[3]
4	$\frac{M_t}{M_\infty} = K.t^n$	Korsmeyer–Peppas	Mt = Amount of drug released at time ttt<br>M∞ = Total amount of drug released at infinite time (i.e., final amount)<br>$\frac{M_t}{M_\infty}$ = Fraction of drug released at time t<br>K = Kinetic constant incorporating structural and geometric characteristics<br>n = Release exponent that indicates the mechanism of drug release	[4]
5	$Q_t = K_H\sqrt{t}$	Higuchi	Q_t = Cumulative amount of drug released at time t<br>K_H=Higuchi dissolution constant (units: amount/time$^{1/2}$)<br>t = Time	[5]

Table S6. The parameter of the kinetic models of DOX release from V-MOF nanofiber membrane

Kinetic model	Value of parameters	
Zero-order	K_o (h)	1.12
	Reduced Chi-Sqr	732.2892
	Residual Sum of Squares	0.94625
	R-Square (COD)	0.89538
	R^2	0.89062
First-order	K_F (h^{-1})	0.39
	Reduced Chi-Sqr	227.04921
	Residual Sum of Squares	0.98205
	R-Square (COD)	0.96443
	R^2	0.96273
Hexson-crowell	K_{HC} (h^{-1})	0.77
	Reduced Chi-Sqr	1507.1648
	Residual Sum of Squares	0.78971
	R-Square (COD)	0.62364
	R^2	0.60653
Kosmeyer-peppas	K_F (h^{-1})	0.12
	n	0.007
	Reduced Chi-Sqr	736.96025
	Residual Sum of Squares	0.94588
	R-Square (COD)	0.8947
	R^2	0.88991
Higuchi	K_H	1.82
	Reduced Chi-Sqr	2086.054
	Residual Sum of Squares	0.9378
	R-Square (COD)	0.863
	R^2	0.858

Table S7. The models have been subjected to analysis of variance.

Source	Sum of squares	df	Mean squares	F-value	P-value	
Model	8266.13	9	918.46	3036.48	< 0.0001	significant
A-pH	350.26	1	350.26	1157.97	< 0.0001	
B-time	7172.51	1	7172.51	23712.79	< 0.0001	
C-Temperature	380.96	1	380.96	1259.49	< 0.0001	
AB	132.41	1	132.41	437.75	< 0.0001	
AC	35.25	1	35.25	116.52	< 0.0001	
BC	157.33	1	157.33	520.15	< 0.0001	
A^2	2.11	1	2.11	6.97	0.0335	
B^2	34.28	1	34.28	113.32	< 0.0001	
C^2	0.3314	1	0.3314	1.10	0.3300	
Residual	2.12	7	0.3025			
Lack of Fit	2.12	3	0.7058			
Pure Error	0.0000	4	0.0000			
Cor Total	8268.24	16				
Std. Dev.	0.5500					
Mean	34.64					
C.V. %	1.59					
R^2	0.9997					
Adjusted R^2	0.9994					
Predicted R^2	0.9959					
Adeq Precision	177.5884					
PRESS	33.88					
-2 Log Likelihood	12.83					
BIC	41.16					
AICc	69.50					

Table S8. Comparison of different nanocarriers based on MOFs for DOX delivery.

Materials	Drug release	Cell lines	Cell viability	Concentration	Treatment	Ref.
LDH-Fe_3O_4/Cu MOF-DOX-CS@CAR<br>CS (chitosan), CAR (carrageenan hydrogel)	pH 5.5 / 72h / 60%<br>pH 7.4 / 72h / 23%	L929<br>MCF-7	95%<br>50%	62.5 µg/mL	48h	[6]
DOX- CS/Fe_3O_4/Cu-MOF<br>CS (chitosan)	pH 4.5 / 96h / 60%<br>pH 7.4 / 96h / 20%	MCF-7	65%	16 µg/mL	48h	[7]
CS/DOX@Ti-MOF<br>Cs (chitosan)	pH 6.5 / 48h / 76%<br>pH 7.4 / 48h / 10%	MNNG/HOS<br>MDA-MB-231	30%<br>20%	6 µg/mL	48h	[8]
SiO2@Fe_3O_4-HA-MIL-100-GQDs-DOX<br>HA (hydroxyapatite), GQDs (graphene quantu0m dots)	pH 5 / 70h / 67%<br>pH 7.4 / 70h / 29%	MCF-7	5%	32 µg/mL	72h	[9]
Alg-DOX-Cu MOF-LDH<br>Alg (alginate)	pH 5 / 72h / 69 %<br>pH 6.8 / 72h / 39%<br>pH 7.4 / 72h / 29%	L929<br>MCF-7	90%<br>10%	60 µg/mL	48h	[10]
UiO-66 @P @ DOX<br>P (porphyrin)	pH 4.5/ 200h /90%<br>pH 5.5 / 200h /70%<br>pH 7.4 / 200h /85%	HEK-293<br>HT-29<br>MCF-7<br>MCF-10A	40%<br>60%<br>20%<br>60%	50 µg/mL	48h	[11]
UiO-66 @P @ DOX@RO<br>P (porphyrin), RO (*Rosmarinus officinalis)*	pH 4.5 / 200h / 40%<br>pH 5.5 / 200h / 60%<br>pH 7.4 / 200h / 50%	HEK-293<br>HT-29<br>MCF-7<br>MCF-10A	80%<br>80%<br>65%<br>80%	50 µg/mL	48h	[11]
A520@L@DOX	pH 4.5/ 200h / 94%<br>pH 5.5 / 200h / 97%<br>pH 7.4 / 200h / 96%	HEK-293<br>HeLa<br>MCF-7<br>PC12	95%<br>65%<br>76%<br>70%	50 µg/mL	48h	[12]
A520@L@DOX@L	pH 4.5 / 150h / 36%<br>pH 5.5 / 150h / 49%<br>pH 7.4 / 150h / 88%	HEK-293<br>HeLa<br>MCF-7<br>PC12	96%<br>90%<br>90%<br>83%	50 µg/mL	48h	[12]

DOX@V-MOF nanofiber membrane	pH 5 / 140h / 96.14%	HepG-2	97.2	107.5 µg/mL	50	This study
	pH 6.2 / 140h / 58.4%	MCF-7	96.4			
	pH 7.4 / 10h / 34.6%					

Table S9. Using different MOFs with different coating agents on different cell lines.

MOFs	Coating agents	Cell lines	Ref.
Silver-Based MOF	Chitosan	L929	[13]
BioMOF	Chitosan	HUVEC	[14]
UiO-66	Fe3O4 Nanoparticles	HeLa, NIH/3T3	[15]
UiO-66	Aloe vera Biopolymer	HFFF2	[16]
UiO-66	PEG	MCF-7	[17]
UiO-68	Aptamer	MDA-MB-23 , MCF-10A	[18]
Cu MOF	L-lysine	MCF-7 , MCF-10A	[19]
Cu MOF	Aptamer	Aptamer	[20]
MIL-100(Fe)	Silica	MCF-7 , MCF-10A	[21]
MIL-100(Fe)	PEG	MCF-7	[22]
ZIF-8	Chitosan & Folic acid	MCF-7	[23]
MIL-88B	Chitosan & Folic acid	M109	[24]
Ni/Ta MOF	Chitosan & Folic acid	MCF-7 , HepG2	[25]
Zn-NMOF	Chitosan & Folic acid	HCT116	[26]
MOF-5	Chitosan & Alginate	HEK-293 , PC12 , HepG2	[27]
MOF-5	Carboxymethylcellulose, Aptamer	HeLa , 4TA	[28]
UiO-66-NH2	Porphyrin	MCF-7 , HT-29	[11]
beta- CD- MOF	Glutamine	MCF-7 , AGS	[29]
Bio-MOF-11	Pectin Biopolymer	SW489	[30]
Fe-BTC MOF	Liposome		[31]
A520	Tp Extract	MCF-7 , HeLa, HEK-293, PC12	[12]
DOX@V-MOF nanofiber membrane	Chitosan and polyvinylalchol	HepG-2, MCF-7	This study

References

[1] K. Ramteke, P. Dighe, A. Kharat, S. Patil, Mathematical models of drug dissolution: A review, Sch. Acad. J. Pharm, 3 (2014) 388-396.

[2] B. Narasimhan, R. Langer, Zero-order release of micro-and macromolecules from polymeric devices: the role of the burst effect, Journal of controlled release, 47 (1997) 13-20.
[3] N. Mulye, S. Turco, A simple model based on first order kinetics to explain release of highly water soluble drugs from porous dicalcium phosphate dihydrate matrices, Drug development and industrial pharmacy, 21 (1995) 943-953.
[4] A. Talevi, M.E. Ruiz, Korsmeyer-Peppas, Peppas-Sahlin, and Brazel-Peppas: Models of drug release, The ADME Encyclopedia: A Comprehensive Guide on Biopharmacy and Pharmacokinetics, Springer2022, pp. 613-621.
[5] D. Paul, Elaborations on the Higuchi model for drug delivery, International journal of pharmaceutics, 418 (2011) 13-17.
[6] A. Taghikhani, M. Babazadeh, S. Davaran, E. Ghasemi, Facile preparation of a pH-sensitive biocompatible nanocarrier based on magnetic layered double hydroxides/Cu MOFs-chitosan crosslinked κ-carrageenan for controlled doxorubicin delivery to breast cancer cells, Colloids and Surfaces B: Biointerfaces, 243 (2024) 114122.
[7] M. Abbasian, M. Khayyatalimohammadi, In-situ forming Cu-based metal-organic framework in the presence of chitosan-Fe_3O_4 nanohybrids: A pH-sensitive carrier for controlled release of doxorubicin, International Journal of Biological Macromolecules, 278 (2024) 134224.
[8] Y. Zeng, J. Yuan, Z. Ran, X. Zhan, X. Li, H. Ye, J. Dong, G. Cao, Z. Pan, Y. Bao, Chitosan/NH_2-MIL-125 (Ti) scaffold loaded with doxorubicin for postoperative bone tumor clearance and osteogenesis: An in vitro study, International Journal of Biological Macromolecules, 263 (2024) 130368.
[9] S. Karimi, V. Zeyni, H. Namazi, A fluorescent system based on graphene quantum dots-capped magnetic hydroxyapatite-MIL-100 metal-organic frameworks for pH-sensitive and controlled release of DOX, Diamond and Related Materials, 140 (2023) 110502.
[10] S. Karimi, H. Rasuli, R. Mohammadi, Facile preparation of pH-sensitive biocompatible alginate beads havening layered double hydroxide supported metal-organic framework for controlled release from doxorubicin to breast cancer cells, International Journal of Biological Macromolecules, 234 (2023) 123538.
[11] S. Ahmadi, V. Jajarmi, M. Ashrafizadeh, A. Zarrabi, J.T. Haponiuk, M.R. Saeb, E.C. Lima, M. Rabiee, N. Rabiee, Mission impossible for cellular internalization: When porphyrin alliance

with UiO-66-NH2 MOF gives the cell lines a ride, Journal of Hazardous Materials, 436 (2022) 129259.
[12] H. Daneshgar, M. Bagherzadeh, S. Sojdeh, M. Safarkhani, M. Edrisi, A. Ojaghi, S. Ahmadi, M. Kiani, N. Rabiee, Discovery of valley-hill structures on the surface of MOFs: Enhancing DOX diffusion and release through nature-made channels, Nano Materials Science, (2024).
[13] S.M. Dehnavi, M. Barjasteh, S.A. Seyedkhani, S.Y. Rahnamaee, R. Bagheri, A novel silver-based metal-organic framework incorporated into nanofibrous chitosan coatings for bone tissue implants, International Journal of Pharmaceutics, 640 (2023) 123047.
[14] R. Abazari, A.R. Mahjoub, F. Ataei, A. Morsali, C.L. Carpenter-Warren, K. Mehdizadeh, A.M. Slawin, Chitosan immobilization on bio-MOF nanostructures: a biocompatible pH-responsive nanocarrier for doxorubicin release on MCF-7 cell lines of human breast cancer, Inorganic Chemistry, 57 (2018) 13364-13379.
[15] H.-X. Zhao, Q. Zou, S.-K. Sun, C. Yu, X. Zhang, R.-J. Li, Y.-Y. Fu, Theranostic metal–organic framework core–shell composites for magnetic resonance imaging and drug delivery, Chemical science, 7 (2016) 5294-5301.
[16] H. Nabipour, S. Rohani, Zirconium metal organic framework/aloe vera carrier loaded with naproxen as a versatile platform for drug delivery, Chemical Papers, 77 (2023) 3461-3470.
[17] V. Gupta, S. Mohiyuddin, A. Sachdev, P. Soni, P. Gopinath, S. Tyagi, PEG functionalized zirconium dicarboxylate MOFs for docetaxel drug delivery in vitro, Journal of Drug Delivery Science and Technology, 52 (2019) 846-855.
[18] W.-H. Chen, X. Yu, A. Cecconello, Y.S. Sohn, R. Nechushtai, I. Willner, Stimuli-responsive nucleic acid-functionalized metal–organic framework nanoparticles using pH-and metal-ion-dependent DNAzymes as locks, Chemical science, 8 (2017) 5769-5780.
[19] M.R. Moghadam, S. Karimi, H. Namazi, A targeted biosystem based on l-lysine coated GO@ rod-Cu (II) metal-organic frameworks for pH-controlled co-delivery of doxorubicin and curcumin, Food Bioscience, 58 (2024) 103578.
[20] M. Falsafi, M. Zahiri, A.S. Saljooghi, K. Abnous, S.M. Taghdisi, A. Sazgarnia, M. Ramezani, M. Alibolandi, Aptamer targeted red blood cell membrane-coated porphyrinic copper-based MOF for guided photochemotherapy against metastatic breast cancer, Microporous and Mesoporous Materials, 325 (2021) 111337.

[21] F. Parsa, M. Setoodehkhah, S.M. Atyabi, Loading and release study of ciprofloxacin from silica-coated magnetite modified by iron-based metal-organic framework (MOF) as a nonocarrier in targeted drug delivery system, Inorganic Chemistry Communications, 155 (2023) 111056.
[22] P. Yadav, S. Kumari, A. Yadav, P. Bhardwaj, M. Maruthi, A. Chakraborty, P. Kanoo, Biocompatible Drug Delivery System Based on a MOF Platform for a Sustained and Controlled Release of the Poorly Soluble Drug Norfloxacin, ACS omega, 8 (2023) 28367-28375.
[23] M. Ghaderpour, S. Kashanian, M. Nazari, M. Motiei, S. Sajadimajd, Targeted Delivery of Letrozole Using a Modified Metal–Organic Framework as a Promising Candidate in Breast Cancer Therapy, BioNanoScience, (2024) 1-14.
[24] S. Dehghani, M. Hosseini, S. Haghgoo, V. Changizi, H. Akbari Javar, M. Khoobi, N. Riahi Alam, Multifunctional MIL-Cur@ FC as a theranostic agent for magnetic resonance imaging and targeting drug delivery: in vitro and in vivo study, Journal of drug targeting, 28 (2020) 668-680.
[25] S.-s. Jalaladdiny, A. Badoei-dalfard, Z. Karami, G. Sargazi, Co-delivery of doxorubicin and curcumin to breast cancer cells by a targeted delivery system based on Ni/Ta core-shell metal-organic framework coated with folic acid-activated chitosan nanoparticles, Journal of the Iranian Chemical Society, 19 (2022) 4287-4298.
[26] Z. Khatibi, N.M. Kazemi, S. Khaleghi, Targeted and biocompatible NMOF as efficient nanocomposite for delivery of methotrexate to colon cancer cells, Journal of Drug Delivery Science and Technology, 73 (2022) 103441.
[27] N. Rabiee, M. Bagherzadeh, M. Jouyandeh, P. Zarrintaj, M.R. Saeb, M. Mozafari, M. Shokouhimehr, R.S. Varma, Natural polymers decorated MOF-MXene nanocarriers for co-delivery of doxorubicin/pCRISPR, ACS applied bio materials, 4 (2021) 5106-5121.
[28] S. Javanbakht, A. Hemmati, H. Namazi, A. Heydari, Carboxymethylcellulose-coated 5-fluorouracil@ MOF-5 nano-hybrid as a bio-nanocomposite carrier for the anticancer oral delivery, International journal of biological macromolecules, 155 (2020) 876-882.
[29] P. Sadeh, S. Zeinali, B. Rastegari, I. Najafipour, Functionalization of β-cyclodextrin metal-organic frameworks with gelatin and glutamine for drug delivery of curcumin to cancerous cells, Heliyon, 10 (2024).
[30] H. Nabipour, Y. Hu, Development of fully bio-based pectin/curcumin@ bio-MOF-11 for colon specific drug delivery, Chemical Papers, 76 (2022) 2969-2979.

[31] A. Karami, A. Ahmed, R. Sabouni, G.A. Husseini, M. Al Sharabati, N. AlSawaftah, V. Paul, Hybrid liposome/metal–organic framework as a promising dual-responsive nanocarriers for anticancer drug delivery, Colloids and Surfaces B: Biointerfaces, 217 (2022) 112599.
